# A mathematical model of cocoa bean fermentation

**DOI:** 10.1098/rsos.180964

**Published:** 2018-10-17

**Authors:** Mauricio Moreno-Zambrano, Sergio Grimbs, Matthias S. Ullrich, Marc-Thorsten Hütt

**Affiliations:** Department of Life Sciences and Chemistry, Jacobs University Bremen, Campus Ring 1, 28759 Bremen, Germany

**Keywords:** theoretical biology, Bayesian parameter estimation, cocoa bean fermentation, microbial fermentation products

## Abstract

Cocoa bean fermentation relies on the sequential activation of several microbial populations, triggering a temporal pattern of biochemical transformations. Understanding this complex process is of tremendous importance as it is known to form the precursors of the resulting chocolate’s flavour and taste. At the same time, cocoa bean fermentation is one of the least controlled processes in the food industry. Here, a quantitative model of cocoa bean fermentation is constructed based on available microbiological and biochemical knowledge. The model is formulated as a system of coupled ordinary differential equations with two distinct types of state variables: (i) metabolite concentrations of glucose, fructose, ethanol, lactic acid and acetic acid and (ii) population sizes of yeast, lactic acid bacteria and acetic acid bacteria. We demonstrate that the model can quantitatively describe existing fermentation time series and that the estimated parameters, obtained by a Bayesian framework, can be used to extract and interpret differences in environmental conditions. The proposed model is a valuable tool towards a mechanistic understanding of this complex biochemical process, and can serve as a starting point for hypothesis testing of new systemic adjustments. In addition to providing the first quantitative mathematical model of cocoa bean fermentation, the purpose of our investigation is to show how differences in estimated parameter values for two experiments allow us to deduce differences in experimental conditions.

## 1. Introduction

**Figure 1.**
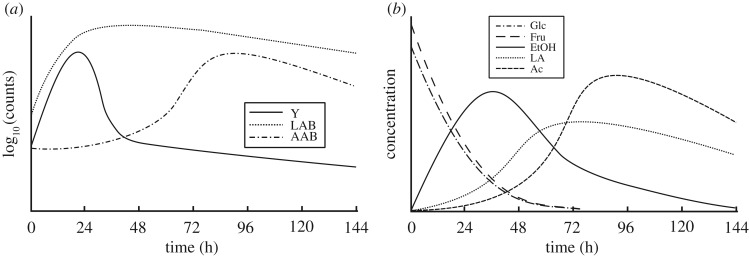
Typical time series for community dynamics and metabolite kinetics during cocoa bean fermentation. (*a*) Community dynamics; yeast (Y), lactic acid bacteria (LAB) and acetic acid bacteria (AAB). (*b*) Metabolite kinetics; glucose (Glc), fructose (Fru), ethanol (EtOH), lactic acid (LA) and acetic acid (Ac). Both counts and concentration are shown in arbitrary units. (After [3]; use permitted under the Creative Commons Attribution License CC-BY 4.0 number 4354810766457).

The fermentation of cocoa beans is recognized as a key step in cocoa processing in terms of the development of chocolate’s flavour and aroma [1,2]. It occurs mainly in the pulp, i.e. a white mucilagenous mass that surrounds the bean, where three major microbial groups drive mostly the whole process whose main activity occurs in a consecutively way (figure 1*a*), being metabolically dominated in earlier stages by yeast (Y) and subsequently surpassed by lactic acid bacteria (LAB), and after the decline of these two first groups, acetic acid bacteria (AAB) take over [4–7]. This so-called three-phase process, depending on the region and local farm practices, is expected to happen within a time frame of 2–10 days [2,3,8–10].

As a result of the fermentation, a series of biochemical reactions are triggered in the raw material, the qualitative characteristics of which have been exhaustively described in terms of the microbial groups involved and the associated metabolic alterations [9,11,12].

Despite the fact that this process is of high industrial relevance, there are hardly any attempts to construct a mathematical model of cocoa bean fermentation. So far, the existing modelling attempts are either focusing on specific post-fermentation steps such as drying kinetics [13,14], restricted to the sequential interaction of microbial communities using metabolic flux analysis [15,16] or kinetic approaches that ‘cannot explain the dynamics in microbial population’ [17].

The reasons for this are manifold, among them, the lacking of control over the fermentation process itself as well as the systemic complexity in terms of involved microbial communities. On the one hand, cocoa bean fermentation is conducted in a spontaneous way unlike the majority of other food fermentation processes [18] with a huge diversity of techniques and devices, e.g. heaps, boxes, baskets, trays, sacks and platforms [2,8,18]. Owing to the lack of control, it is difficult to identify the crucial parameters and key variables required for the formulation of an appropriate model.

On the other hand, in contrast to other relevant industrial fermentation processes such as those of beer and wine, the fermentation in cocoa involves microbial community dynamics of three major microbial groups, i.e. Y, LAB and AAB, which are, in turn, represented by several different strains [3,4,6,7]. Hence, the complexity is precisely one of the biggest challenges to overcome since growth modelling of microorganisms has been classically applied under much more controlled conditions that involve single strain cultures where mortality phenomena have been scarcely considered [19]. Consequently, this needs to be taken into account for an approximation of the cocoa bean fermentation process.

Cocoa bean fermentation is a prototypical situation for the application of modelling using coupled nonlinear ordinary differential equations (ODE): The initial situation displays a rich diversity with a multitude of influencing factors and the result of the dynamic process, the fermented cocoa bean, is of high relevance for the subsequent industrial processing steps and for the quality of the final product, chocolate.

Here, we present a one-compartment model for the cocoa bean fermentation process using the mathematical concepts of the Monod [20] and Contois [21] equations, assuming that single strain kinetic modelling techniques can describe the growth of mixtures of microbial species belonging to different microbial groups in the same environment. Moreover, microbial death processes are handled by the use of the Chick–Watson mortality law [22].

While conceptionally the modelling approach presented here is rather in the tradition of theoretical biology, the way to analyse and apply the model differs from e.g. a traditional linear stability analysis, as the purpose of the model is predominantly to describe *transients* in a batch culture [23], rather than asymptotic states as would be expected in continuous cultures.

With the model constructed along these lines, we were able to describe three datasets corresponding to two different cocoa-producing countries where two different fermentation methodologies were implemented. In that way; our model also can interpret differences in the experimental set-up of the two trials conducted under the same methodology, in terms of significant changes in the estimated parameters. This approach serves as a source of elucidation of possible hypotheses on how these parameters are affected by slight changes within a particular region where the fermentation took place.

## 2. Material and methods

### 2.1. Experimental data

The experimental data used in this study were reported in Camu *et al.* [4] and Papalexandratou *et al.* [24]. In both instances, the predominant cocoa hybrids harnessed by the chocolate industry, Criollo and Forastero, were used as the source of raw material. In the study of Camu *et al.* [4], the beans were fermented by the heaps method, while for Papalexandratou *et al.* [24], wooden boxes were used as fermenting devices. The data of Camu *et al.* [4] were collected in Ghana from seven trials in two field experiments and data of one representative trial were published. The data include measurements of microbial counts of Y, LAB, AAB and total aerobic bacteria. Metabolite time series measured both in pulp and bean are available for glucose (Glc), fructose (Fru), sucrose, lactic acid (LA), acetic acid (Ac), ethanol (EtOH), mannitol, citric acid and succinic acid.

The data reported by Papalexandratou *et al.* [24] were collected in Brazil from two trials in two field experiments, of which both trials were published as ‘box 1’ and ‘box 2’. The conditions in which both trials were conducted differed slightly. On the one hand, the fermenting mass of box 1 was placed under a metal roof to protect it from weather conditions. On the other hand, the fermentation for box 2 was carried out in a fermentary room. The data include measurements of microbial counts of Y, LAB, AAB and total aerobic bacteria. Metabolite time series measured both in pulp and bean are available for Glc, Fru, sucrose, LA, Ac, EtOH, mannitol and gluconic acid.

In both collections, the fermentation trials took place in a time frame of 6 days with measurements performed at 17 time points for Camu *et al.* [4] and 14 time points for Papalexandratou *et al.* [24]. Abiotic factors, i.e. temperature and pH, were measured as well. Cell counts were done by means of malt extract agar, Man–Rogosa–Sharpe agar and deoxycholate-mannitol-sorbitol agar for Y, LAB and AAB, respectively, from both data sources. As metabolite time series, we considered Glc, Fru, EtOH, LA and Ac.

### 2.2. Microbial count units transformation

One of the most common forms of quantifying microbial growth is the count of colony forming units (CFU), specially when dealing with mixtures of microorganisms as the microbial successions are reported in the original works of Camu *et al.* [4] and Papalexandratou *et al.* [24]. In this sense, the vast majority of studies involving single-strained microbial growth are reported in terms of dry biomass as well as their dependent constants, i.e. maximum growth rates, mortality rates and yield coefficients.

In order to obtain comparable estimates to those available for species of these microbial groups in the literature, a conversion from CFU to dry biomass units was conducted based on available knowledge as well as geometric deductions for the microbial group involved in the process.

For species within the microbial group of Y, we used the conversion factor that one CFU of *S. cerevisiae* is equivalent to 15 picograms (pg), as assumed by Schwabe & Bruggeman [25]. For LAB and AAB, since such conversion factors have not been reported yet, values were inferred by taking into account a geometric approximation based on the usual dimensions of the cells belonging to the genus of *Acetobacter* and to the species of *Lactobacillus plantarum*, respectively, according to the *Bergey’s Manual of Systematic Bacteriology* [26,27] and assuming their shape given by a spherocylinder. Thus, using as reference a density value derived from the dry weight of a cell of *E. coli* of 0.28 pg [28] per micro cubic metre (μm^3^) [29], the conversion factor between CFU to dry biomass of LAB and AAB were determined as 1.25 and 0.28 pg CFU^−1^, respectively (see electronic supplementary material, S1).

### 2.3. Model development

#### 2.3.1. Biochemical background

**Figure 2.**
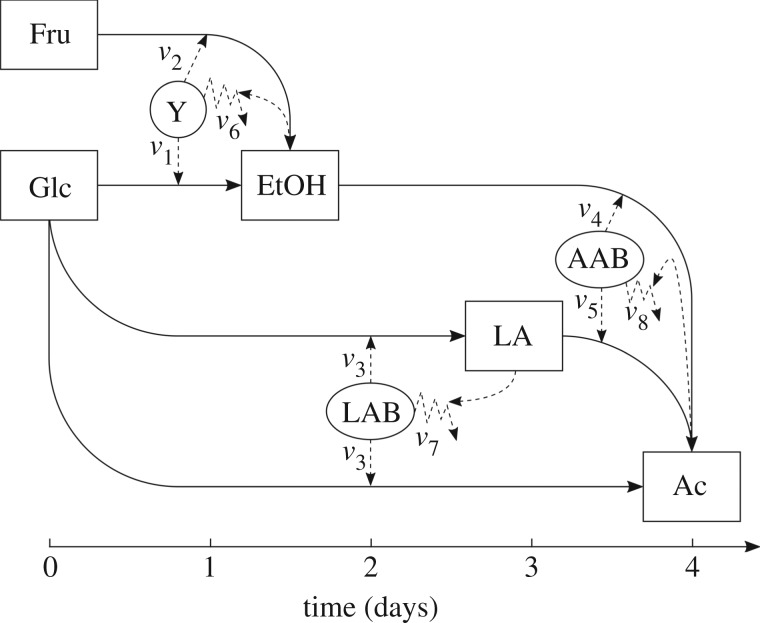
Network diagram of the cocoa bean fermentation model. Microbial groups: yeast (Y), lactic acid bacteria (LAB) and acetic acid bacteria (AAB) are represented as circles. Metabolites: glucose (Glc), fructose (Fru), ethanol (EtOH), lactic acid (LA) and acetic acid (Ac) are represented as squares. The growth rates of yeast on glucose (*v*_1_) and fructose (*v*_2_), of lactic acid bacteria (*v*_3_), and of acetic acid bacteria on ethanol (*v*_4_) and acetic acid (*v*_5_) are represented as straight dashed arrows. The mortality rates of yeast (*v*_6_), lactic acid bacteria (*v*_7_) and acetic acid bacteria (*v*_8_) are represented as zigzag dashed arrows. Straight dashed arrows pointing from products to mortality rates represent product influence on mortality rates. Solid straight arrows show the direction in which the conversion of metabolites occurs.

The fermentation of cocoa beans has been described in detail with respect to its microbial dynamics and metabolite kinetics in both pulp and bean [3,4,6–8]. From such descriptions, the fermentation process in cocoa can be understood as an overlapping succession of microbial activities that mostly occur in the pulp, where three core processes are easily identifiable. These are the conversion of Glc and Fru into EtOH by Y, Glc into LA by LAB and EtOH into Ac by AAB (figure 1). Further processes such as the conversions of Glc into Ac by LAB and LA into Ac by AAB, have also been described [30]. The interpretation of these processes in a network diagram covering the pulp only, is shown in figure 2, where microbial growth rate is taken into account represented as the uptake of the respective substrates as well as the mortality rates for Y, LAB and AAB.

In our model, we consider a simultaneous growth of the three major microbial groups. The sequential dominance in the process is then emerging from the availability pattern of their respective substrates without taking into account abiotic factors.

#### 2.3.2. Mathematical representation

The process of cocoa bean fermentation can be seen as a batch process that can involve the usual phases of microbial growth, i.e. lag, exponential, stationary and death phase. However, it is known that microbial growth occurring in natural environments might show different patterns [31]. In this sense, the two collections of experimental data on microbial successions expressed as the log CFU showed basically phases that resembled the exponential and death phases without noticeable stationary or lag phases. From the mathematical perspective, such phenomena can be expressed as an ODE for each of the state variables involved in such a way that the growth of microorganisms is dependent on the availability of their respective substrates, together with mortality equations to capture the inherent decay of the populations along time.

The two major effects on population size considered here, exponential growth and death phase, were modelled by different approaches. On the one hand, we use the classical Monod [20] and Contois [21] equations to describe the growth of groups of microorganisms belonging to a same microbial group (namely, Y, LAB and AAB), instead of the common use of these terms for single strain cultures. Accordingly, the growth rates of Y, *v*_1_ on Glc and *v*_2_ on Fru, of LAB, *v*_3_, and of AAB on EtOH, *v*_4_, (shown in figure 2) have the form of Monod equations, while the growth of AAB on LA, *v*_5_, is a Contois equation. The use of a Contois term for *v*_5_ was considered under the assumption that, given that few species of AAB are capable of catabolizing lactic acid [16,32], the growth rate of these species is a function of their population size (see electronic supplementary material, S2). On the other hand, the mortality rates of all microbes, *v*_6_, *v*_7_ and *v*_8_, are modelled as Chick–Watson equations [22] considering a nonlinear decay of microbial populations produced by second- and third-order reactions of their corresponding metabolite products upon themselves, as shown in table 1. Together, all equations in table 1 comprise 13 parameters: (i) five maximum specific growth rates, (ii) five substrate saturation constants, and (iii) three mortality rate constants.

**Table 1.** Growth and mortality rate equations for the cocoa bean fermentation process. Microbial groups are represented as yeast (Y), lactic acid bacteria (LAB) and acetic acid bacteria (AAB). Metabolites are represented as glucose (Glc), fructose (Fru), ethanol (EtOH), lactic acid (LA) and acetic acid (Ac). Biomass and concentration of metabolites are both shown within square brackets [ ]. Maximum specific growth rates *μ*_max_ are shown of the form $\mu_{\max}^{i_{n}}$, where *i* can be either Y, LAB and AAB, and *n* refers to whether *μ* corresponds to the maximum specific growth of Y on either Glc or Fru, or AAB on either EtOH or LA. Substrate saturation constants for the growth of Y, LAB and AAB are shown of the form $K_{m}^{\;j}$, where *j* can be either Y or LAB and *m* can be either Glc, Fru, EtOH and LA. Constant mortality rates are shown of the form *k*_*i*_, where *i* can be either Y, LAB or AAB.

growth rate equation	mortality rate equation
$\begin{array}{rl} v_{1} & =\frac{\mu_{\max}^{Y_{\mathrm{Glc}}}\;[\text{Glc}]}{[\text{Glc}]+K_{\mathrm{Glc}}^{Y}}\;[\text{Y}]\\ v_{2} & =\frac{\mu_{\max}^{Y_{\mathrm{Fru}}}\;[\text{Fru}]}{[\text{Fru}]+K_{\mathrm{Fru}}^{Y}}\;[\text{Y}] \end{array}$	$\begin{array}{rl} v_{6} & =k_{Y}\;[\text{Y}]\;[\text{EtOH}] \end{array}$
$\begin{array}{rl} v_{3} & =\frac{\mu_{\max}^{\mathrm{LAB}}\;[\text{Glc}]}{[\text{Glc}]+K_{\mathrm{Glc}}^{\mathrm{LAB}}}\;[\text{LAB}] \end{array}$	$\begin{array}{r} v_{7}=k_{\mathrm{LAB}}\;[\text{LAB}]\;[\text{LA}] \end{array}$
$\begin{array}{rl} v_{4} & =\frac{\mu_{\max}^{{\mathrm{AAB}}_{\mathrm{EtOH}}}\;[\text{EtOH}]}{[\text{EtOH}]+K_{\mathrm{EtOH}}^{\mathrm{AAB}}}\;[\text{AAB}]\\ v_{5} & =\frac{\mu_{\max}^{{\mathrm{AAB}}_{\mathrm{LA}}}\;[\text{LA}]}{\left[\text{LA}]+K_{\mathrm{LA}}^{\mathrm{AAB}}\;[\text{AAB}\right]}[\text{AAB}] \end{array}$	$\begin{array}{r} v_{8}=k_{\mathrm{AAB}}\;[\text{AAB}]\;{\left[\text{Ac}\right]}^{2} \end{array}$

From the set of growth and mortality rate equations defined in table 1, a system of ODEs can be established in order to mathematically express the given network considering the necessary eleven yield coefficients to take into account the amounts of biomass that can be obtained from substrate as well as the amounts of produced metabolites, as shown in the system of ODEs in equations (2.1) to (2.8) that represent Glc, Fru, EtOH, LA, Ac, Y, LAB and AAB, respectively. A complete interpretation of the 24 estimated parameters is given in table 2.

**Table 2.** Parameters of the cocoa bean fermentation model and their interpretation. Microbial groups: yeast (Y), lactic acid bacteria (LAB) and acetic acid bacteria (AAB). Metabolites: glucose (Glc), fructose (Fru), ethanol (EtOH), lactic acid (LA) and acetic acid (Ac).

parameter	unit	interpretation
$\mu_{\max}^{Y_{\mathrm{Glc}}}$	h^−1^	maximum specific growth rate of Y on Glc
$\mu_{\max}^{Y_{\mathrm{Fru}}}$	h^−1^	maximum specific growth rate of Y on Fru
$\mu_{\max}^{\mathrm{LAB}}$	h^−1^	maximum specific growth rate of LAB on Glc
$\mu_{\max}^{{\mathrm{AAB}}_{\mathrm{EtOH}}}$	h^−1^	maximum specific growth rate of AAB on EtOH
$\mu_{\max}^{{\mathrm{AAB}}_{\mathrm{LA}}}$	h^−1^	maximum specific growth rate of AAB on LA
$K_{\mathrm{Glc}}^{Y}$	mg(Glc) g(pulp)^−1^	substrate saturation constant of Y growth on Glc
$K_{\mathrm{Fru}}^{Y}$	mg(Fru) g(pulp)^−1^	substrate saturation constant of Y growth on Fru
$K_{\mathrm{Glc}}^{\mathrm{LAB}}$	mg(Glc) g(pulp)^−1^	substrate saturation constant of LAB growth on Glc
$K_{\mathrm{EtOH}}^{\mathrm{AAB}}$	mg(EtOH) g(pulp)^−1^	substrate saturation constant of AAB growth on EtOH
$K_{\mathrm{LA}}^{\mathrm{AAB}}$	mg(LA) g(pulp)^−1^	substrate saturation constant of AAB growth on LA
*k*_Y_	mg(EtOH)^−1^ h^−1^	mortality rate constant of Y
*k*_LAB_	mg(LA)^−1^ h^−1^	mortality rate constant of LAB
*k*_AAB_	mg(Ac)^−2^ h^−1^	mortality rate constant of AAB
*Y*_Glc | Y_	mg(Glc) mg(Y)^−1^	Y-to-Glc yield coefficient
*Y*_Glc | LAB_	mg(Glc) mg(LAB)^−1^	LAB-to-Glc yield coefficient
*Y*_Fru | Y_	mg(Fru) mg(Y)^−1^	Y-to-Fru yield coefficient
$Y_{\mathrm{EtOH}\;|\;Y}^{\mathrm{Glc}}$	mg(EtOH) mg(Y)^−1^	Y-to-EtOH from Glc yield coefficient
$Y_{\mathrm{EtOH}\;|\;Y}^{\mathrm{Fru}}$	mg(EtOH) mg(Y)^−1^	Y-to-EtOH from Fru yield coefficient
*Y*_EtOH | AAB_	mg(EtOH) mg(AAB)^−1^	AAB-to-EtOH yield coefficient
*Y*_LA | LAB_	mg(LA) mg(LAB)^−1^	LAB-to-LA yield coefficient
*Y*_LA | AAB_	mg(LA) mg(AAB)^−1^	AAB-to-LA yield coefficient
*Y*_Ac | LAB_	mg(Ac) mg(LAB)^−1^	LAB-to-Ac yield coefficient
$Y_{\mathrm{Ac}\;|\;\mathrm{AAB}}^{\mathrm{EtOH}}$	mg(Ac) mg(AAB)^−1^	AAB-to-Ac from EtOH yield coefficient
$Y_{\mathrm{Ac}\;|\;\mathrm{AAB}}^{\mathrm{LA}}$	mg(Ac) mg(AAB)^−1^	AAB-to-Ac from LA yield coefficient

2.1 $$\frac{\text{d}[\text{Glc}]}{\text{d}t}=-Y_{\mathrm{Glc}\;|\;Y}v_{1}-Y_{\mathrm{Glc}\;|\;\mathrm{LAB}}v_{3},$$

2.2 $$\frac{\text{d}[\text{Fru}]}{\text{d}t}=-Y_{\mathrm{Fru}\;|\;Y}v_{2},$$

2.3 $$\frac{\text{d}[\text{EtOH}]}{\text{d}t}=Y_{\mathrm{EtOH}\;|\;Y}^{\mathrm{Glc}}v_{1}+Y_{\mathrm{EtOH}\;|\;Y}^{\mathrm{Fru}}v_{2}-Y_{\mathrm{EtOH}\;|\;\mathrm{AAB}}v_{4},$$

2.4 $$\frac{\text{d}[\text{LA}]}{\text{d}t}=Y_{\mathrm{LA}\;|\;\mathrm{LAB}}v_{3}-Y_{\mathrm{LA}\;|\;\mathrm{AAB}}v_{5},$$

2.5 $$\frac{\text{d}[\text{Ac}]}{\text{d}t}=Y_{\mathrm{Ac}\;|\;\mathrm{LAB}}v_{3}+Y_{\mathrm{Ac}\;|\;\mathrm{AAB}}^{\mathrm{EtOH}}v_{4}+Y_{\mathrm{Ac}\;|\;\mathrm{AAB}}^{\mathrm{LA}}v_{5},$$

2.6 $$\frac{\text{d}[\text{Y}]}{\text{d}t}=v_{1}+v_{2}-v_{6},$$

2.7 $$\frac{\text{d}[\text{LAB}]}{\text{d}t}=v_{3}-v_{7}$$

2.8 $$\;\mathrm{and}\;\frac{\text{d}[\text{AAB}]}{\text{d}t}=v_{4}+v_{5}-v_{8}.$$

The proposed model (as described in equations (2.1)–(2.8)) relies on three simple general assumptions: (i) relationships between Y and AAB, as well as of LAB and AAB, are of a pure commensalistic nature since there is no competition between them for any substrate, i.e. Glc and Fru, and there is no direct effect upon the growth either of Y or LAB by the uptake of its main products, LA and EtOH, by AAB, respectively [33]; (ii) relationship between Y and LAB is a resource-type competition because both microbial groups share Glc as a main limiting substrate and they do not excrete metabolites affecting each other’s growth [34,35]; and (iii) no impact of chemical and physical effects such as temperature and pH on the set of kinetic parameters.

### 2.4. Parameter estimation

#### 2.4.1. Bayesian framework

In order to estimate the 24 parameters of the proposed model, a Bayesian framework was considered by fitting it to the experimental data. In this way, these parameters can be composed in a vector *θ* of the form [*θ*_1_, *θ*_2_, … , *θ*_*k*_]. If it is assumed that *θ* has given rise to the data D, the problem can be solved by inferring the posterior probability of *θ* given D, P(θ∣D).

Assuming that there exist *T* time series in a model, with *N* independently measured data points at time *j* each, the posterior probability (P(θ∣D)) can be expressed as the product over all series and each data point within time series as:

2.9 $$P(\theta |D)\propto \prod_{i=1}^{T}\prod_{\;j=1}^{N}P(D_{i,j}|\theta )P(\theta ),$$

where $D_{i,j}$ is the data point from time series *i* measured at time *j*.

The deterministic model proposed in equations (2.1)–(2.8), can take the general form:

2.10 $$\frac{\text{d}x_{i}}{\text{d}t}=f(x_{i,j},\theta ),$$

where *x*_*i*_ denotes each of the state variables and *f*(*x*_*i*,*j*_, *θ*) the model prediction of the change of *x*_*i*_ at time *j* as a function of the parameter vector *θ*. Each experimental observation ($D_{i,j}$) can be considered as drawn from a normal distribution whose mean is equal to the model prediction *f*(*x*_*i*,*j*_, *θ*), with a necessary standard deviation, σ, that accounts for both experimental measurement error of the real observations and misprediction of the model. In this framework, each observation $D_{i,j}$ is drawn from a sampling distribution of the form

2.11 $$D_{i,j}∼N(f(x_{i,j},\theta ),\sigma ),$$

thus allowing to reformulate the total posterior probability distribution in equation (2.9) as

2.12 $$P(\theta |D)\propto \prod_{i=1}^{T}\prod_{\;j=1}^{N}N(f(x_{i,j},\theta ),\sigma )P(\theta ).$$

Using this form of the total posterior probability, in addition to the 24 parameters from equations (2.1) to (2.8), the total standard deviation, σ, has also been estimated.

#### 2.4.2. Variable scaling

For both collections of experimental data, the concentrations of microorganisms and metabolites differ by several orders of magnitude. As an example, after the transformation of CFU to biomass units in the experimental data of Camu *et al.* [4], the maximum concentration of AAB is approximately 0.0019 mg g(pulp)^−1^, while the maximum concentration of the main substrate of AAB, EtOH, is approximately 22.4920 mg g(pulp)^−1^.

These different orders of magnitude between the state variables can lead to numerical issues during optimization. In order to reduce such issues, all state variables were scaled by dividing each of the time series in the experimental data by its own maximum value. Consequently, possible large differences between the parameters to be estimated are avoided and, most importantly, the search space can be constrained.

Hence, in a first step, the parameters were estimated using time series with a maximum value of 1 and, in a second step, re-scaled to their original physical units through conversion factors derived from equations (2.1) to (2.8) (see electronic supplementary material, S3).

#### 2.4.3. Priors

By scaling the system to allow maximum values for each time series equal to unity, the large differences in orders of magnitude between the parameter estimates, e.g. maximum specific growth rates in the boundaries of fractions of milligrams with respect to yield coefficients that might take values of hundreds, are regularized; in this case, by introducing a scale that needs a prior distribution to be sampled within values between 0 and 1. In that way, an independent normal distribution with mean 0.5 and a standard deviation of 0.3 as prior choice for each *k* element of *θ* represents a weakly informative prior by introducing scale information of the original units in which the parameters of the model are originally measured. For the standard deviation σ, a Cauchy distribution C with location and scale parameters of 0 and 1, respectively, was used as prior distribution. This choice follows the same reasoning as depicted for the *k* independent priors for *θ*, with the addition that the heavy tails of C allow for the sampling of extreme values which would account for outlying observations in the original data. To avoid the estimation of negative parameters, both priors are constrained to take values in the positive set of real numbers and are mathematically expressed as

2.13 $$\left.\begin{array}{rl} \theta_{k} & ∼N(0.5,0.3),\;\theta_{k}>0\\ \;\text{and}\;\sigma & ∼C(0,1),\;\sigma >0. \end{array}\right\}$$

#### 2.4.4. Implementation

The Bayesian parameter estimation framework was performed with Stan [36], using the RStan interface package for R [37,38]. The model was solved as an inital value problem, where the initial concentrations for the eight state variables in equations (2.1) to (2.8) were provided as they were reported in the original works of Camu *et al.* [4] and Papalexandratou *et al.* [24]. Sampling for obtaining the posterior distributions of the unknown parameters as well as the model predictions was conducted using full Bayesian inference through the Markov chain Monte Carlo (MCMC) No-U-Turn sampler method [39]. The ODEs were specified and solved by the built-in mechanism of Stan *rk45*, which provides a fourth- and fifth-order Runge–Kutta method for solving non-stiff systems [40,41]. All datasets were fitted by running four parallel Markov Chains of 3000 iterations each, 1000 of which were used as warm-up. Convergence of the sampling was determined by examining the $\hat{R}$ statistics computed by Stan.

### 2.5. Statistical analyses

Once the parameters of equations (2.1)–(2.8) were estimated, their posterior distributions obtained from fitting the model to each of the two trials, i.e. box 1 and box 2, reported by Papalexandratou *et al.* [24] were compared between each other. For doing so, an effect size statistic was used as proposed by Cohen [42], as a measure of the magnitude of either their relationship or difference. In that way, the effect size expressed as the standardized mean difference (*d*) of two independent continuous distributions was computed as

2.14 $$d=\frac{{\bar{\theta }}_{k,1}-{\bar{\theta }}_{k,2}}{S_{w_{k}}},$$

where ${\bar{\theta }}_{k,1}$ and ${\bar{\theta }}_{k,2}$ are the sampled means corresponding to the parameter *k* obtained from box 1 and box 2, respectively, and $S_{w_{k}}$ is the pooled within-groups standard deviation corresponding to the parameter *k*, which for the case of groups of equal sample sizes (in this study represented as the number of iterations of the MCMC sampler) is given by

2.15 $$S_{w_{k}}=\sqrt{\frac{S_{k,1}^{2}+S_{k,2}^{2}}{2}},$$

where *S*_*k*,1_ and *S*_*k*,2_ are the standard deviations of the posterior distribution of parameter *k* for box 1 and box 2, respectively. We used a threshold of |*d*| > 1.2, in order to identify significant differences between parameters [43].

## 3. Results

### 3.1. Model’s diagnostics

In all three datasets, the proposed model was fitted without major issues. The calculated $\hat{R}$ statistic was 1 for all three cases (see electronic supplementary material, tables S4–S6), showing that convergence of the MCMC sampler was accomplished. Such a behaviour is also noticeable in the obtained traceplots (see electronic supplementary material, figures S6–S8), that show the typical ‘caterpillar’ shape as probe of a good mixing of the MCMC sampler along the exploration of the parameter space. Asymptotically, the ODE system converges to a stable fixed point (see electronic supplementary material, S5).

### 3.2. Metabolite and microbial population dynamics

**Figure 3.**
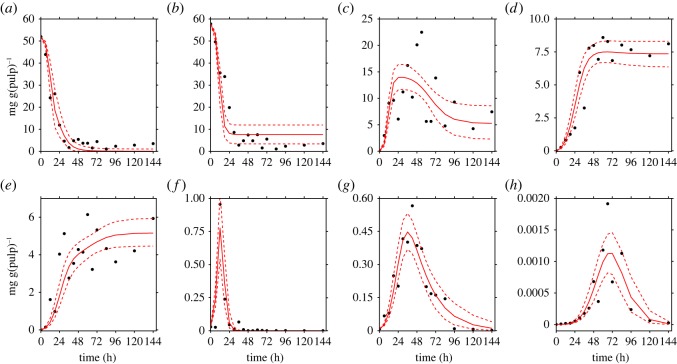
Simulation results of the cocoa bean fermentation model for the data reported by Camu *et al.* [4]. Metabolites: (*a*) glucose, (*b*) fructose, (*c*) ethanol, (*d*) lactic acid and (*e*) acetic acid. Microbial groups: (*f*) yeast, (*g*) lactic acid bacteria and (*h*) acetic acid bacteria. Solid red lines show the simulations of the model, while black points denote the experimental data of Camu *et al.* [4]. The red dashed lines represent the 95% credible interval of the model predictions.

**Figure 4.**
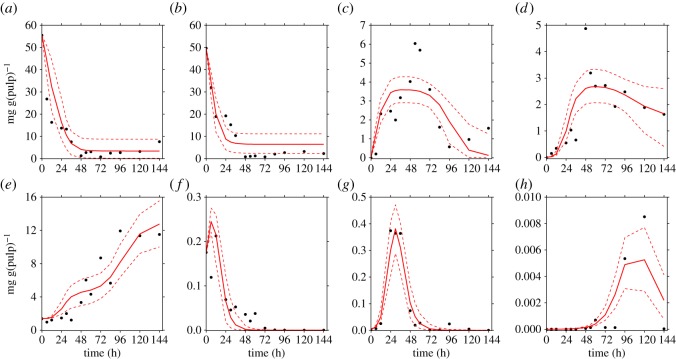
Simulation results of the cocoa bean fermentation model for the data reported for box 1 by Papalexandratou *et al.* [24]. Metabolites: (*a*) glucose, (*b*) fructose, (*c*) ethanol, (*d*) lactic acid and (*e*) acetic acid. Microbial groups: (*f*) yeast, (*g*) lactic acid bacteria and (*h*) acetic acid bacteria. Solid red lines show the simulations of the model, while black points denote the experimental data for box 1 of Papalexandratou *et al.* [24]. The red dashed lines represent the 95% credible interval of the model predictions.

**Figure 5.**
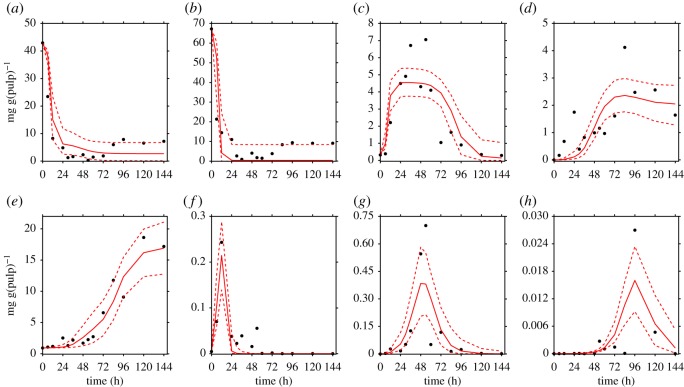
Simulation results of the cocoa bean fermentation model for the data reported for box 2 by Papalexandratou *et al.* [24]. Metabolites: (*a*) glucose, (*b*) fructose, (*c*) ethanol, (*d*) lactic acid and (*e*) acetic acid. Microbial groups: (*f*) yeast, (*g*) lactic acid bacteria and (*h*) acetic acid bacteria. Solid red lines show the simulations of the model, while black points denote the experimental data for box 2 of Papalexandratou *et al.* [24]. The red dashed lines represent the 95% credible interval of the model predictions.

The proposed model, as described in equations (2.1)–(2.8), fits each of the collections of data reported previously by Camu *et al.* [4] (figure 3) and Papalexandratou *et al.* [24] (figures 4 and 5) remarkably well.

In each of the data collections, despite the noisy nature of the experimental data and the low sampling rate, the corresponding simulations show the microbial succession previously reported by several studies for Y, LAB and AAB that emerges from the interplay of metabolites and microbial communities.

Thus, most of the experimental observations reported by Camu *et al.* [4] and Papalexandratou *et al.* [24] fall within the computed 95% credible intervals of the simulations indicating that the model is capable of predicting the dynamics of metabolites in all cases, even in those where theoretical knowledge is not fully reflected on the data. This is the case for the time series of LA in the data reported by Camu *et al.* [4], where its concentrations seem to stay steady after 48 h of fermentation, which would contradict its consumption by AAB as reported by Pereira *et al.* [30].

### 3.3. Parameter estimates

It was found that among the 24 parameters of the model, there are similar reported values for 14 of them for single strain cultures. This means that in our parameter estimation framework, for each dataset 14 parameter estimates can be compared with their counterparts in the literature. In this way, from a total of 42 parameter estimates, 17 (40.48%) of them are in accordance to the referenced ranges, 20 (47.62%) are out of the referenced range by less than the estimate divided or multiplied by 10, and 5 (11.90%) are out of the referenced range by more than the estimate divided or multiplied by 10 (table 3). In the following paragraphs, our results will be structured in accordance to their type as (i) maximum specific growth rates, (ii) substrate saturation constants, (iii) mortality rates, and (iv) yield coefficients.

**Table 3.** Parameter estimates of the cocoa bean fermentation model using a Bayesian estimation framework. Means and standard deviations (in parenthesis) of the posterior distributions for the data of Camu *et al.* [4] and the fermentation boxes 1 and 2 of Papalexandratou *et al.* [24] are shown in columns Camu, P. box 1 and P. box 2, respectively. Additionally, the absolute value of the standardized mean difference (effect size |*d*|) between the estimates of P. box 1 and P. box 2 is shown. Effect sizes marked with (*) are significant (|*d*| > 1.2). Reported values in the literature have same units as shown in table 2, except for the substrate saturation constants, which are given in milligrams of substrate per millilitre of medium (mg(substrate) ml^−1^). Green, orange and red coloured cells correspond to parameter estimates that lie within the referenced range, are out of the range by less than the estimate divided or multiplied by 10 and are out of the range by more than the estimate divided or multiplied by 10, respectively.

parameter	Camu	P. box 1	P. box 2	effect size |*d*|	reported values	reference
$\mu_{\max}^{Y_{\mathrm{Glc}}}$	0.253 (0.094)	0.063 (0.025)	0.368 (0.131)	3.23*	0.0781–0.53	[44–51]
$\mu_{\max}^{Y_{\mathrm{Fru}}}$	0.359 (0.106)	0.083 (0.03)	0.572 (0.151)	4.49*	0.01–0.166	[49,51–53]
$\mu_{\max}^{\mathrm{LAB}}$	0.358 (0.067)	0.414 (0.096)	0.499 (0.152)	0.67	0.0072–1.41	[54–59]
$\mu_{\max}^{{\mathrm{AAB}}_{\mathrm{EtOH}}}$	0.380 (0.092)	0.150 (0.051)	0.168 (0.052)	0.35	0.0106–0.25	[60–64]
$\mu_{\max}^{{\mathrm{AAB}}_{\mathrm{LA}}}$	0.008 (0.012)	0.025 (0.017)	0.022 (0.016)	0.18	n.a.	n.a.
$K_{\mathrm{Glc}}^{Y}$	35.322 (13.826)	34.366 (14.964)	30.015 (10.887)	0.33	9.73 × 10^−4^–0.5	[46,48]
$K_{\mathrm{Fru}}^{Y}$	35.492 (15.253)	25.015 (14.187)	41.386 (17.259)	1.04	582–772	[53]
$K_{\mathrm{Glc}}^{\mathrm{LAB}}$	37.966 (12.37)	31.664 (13.882)	19.272 (10.599)	1.00	0.790–178.0	[57–59]
$K_{\mathrm{EtOH}}^{\mathrm{AAB}}$	16.056 (5.646)	3.818 (1.637)	4.056 (1.98)	0.13	n.a.	n.a.
$K_{\mathrm{LA}}^{\mathrm{AAB}}$	2509.622 (1234.114)	312.051 (153.064)	81.981 (41.365)	2.05*	n.a.	n.a.
*k*_Y_	0.0333 (0.0051)	0.0517 (0.0119)	0.092 (0.0248)	2.07*	n.a.	n.a.
*k*_LAB_	0.0054 (0.0014)	0.0637 (0.0183)	0.0686 (0.025)	0.22	n.a.	n.a.
*k*_AAB_	0.0069 (0.0014)	0.0004 (0.0002)	0.0004 (0.0002)	0.00	n.a.	n.a.
*Y*_Glc | Y_	33.400 (11.255)	240.926 (59.899)	119.672 (35.155)	2.47*	1.56–66.67	[44–46,50,51,65,66]
*Y*_Glc | LAB_	29.259 (10.852)	20.217 (13.24)	3.323 (3.417)	1.75*	4.50–200	[59,67]
*Y*_Fru | Y_	41.105 (11.215)	244.153 (46.219)	232.383 (62.958)	0.21	43.48–200	[51,52]
$Y_{\mathrm{EtOH}\;|\;Y}^{\mathrm{Glc}}$	7.436 (4.526)	11.941 (6.061)	8.201 (4.503)	0.70	1.39–21.49	[46,49,50,68]
$Y_{\mathrm{EtOH}\;|\;Y}^{\mathrm{Fru}}$	5.927 (3.351)	11.195 (5.005)	6.008 (3.882)	1.16	5.7878	[49]
*Y*_EtOH | AAB_	1298.070 (637.461)	378.452 (152.674)	170.44 (56.123)	1.81*	8.06–166.67	[61,63,69]
*Y*_LA | LAB_	10.617 (1.54)	2.785 (0.664)	2.138 (0.799)	0.88	n.a.	n.a.
*Y*_LA | AAB_	1928.619 (1216.393)	287.511 (141.381)	62.9 (38.531)	2.17*	7.94	[63]
*Y*_Ac | LAB_	5.612 (0.919)	3.279 (1.125)	2.899 (1.898)	0.24	n.a.	n.a.
$Y_{\mathrm{Ac}\;|\;\mathrm{AAB}}^{\mathrm{EtOH}}$	104.056 (98.355)	576.471 (271.434)	321.858 (132.72)	1.19	n.a.	n.a.
$Y_{\mathrm{Ac}\;|\;\mathrm{AAB}}^{\mathrm{LA}}$	1427.225 (865.061)	666.903 (357.131)	385.172 (177.474)	1.00	n.a.	n.a.
σ	0.149 (0.010)	0.168 (0.014)	0.168 (0.013)	0.00	n.a.	n.a.

#### 3.3.1. Maximum specific growth rates

With respect to the maximum specific growth rates, the estimated values mostly fall within their reported values in the literature, with the exception of three estimates belonging to particular trials. Specifically, for the maximum specific growth rate of Y on Glc ($\mu_{\max}^{Y_{\mathrm{Glc}}}$), the obtained parameter values ranged from 0.06 to 0.37 h^−1^. These values agree with the reported ones for species of this microbial group, between 0.0781 and 0.53 h^−1^ [44–51] for Camu *et al.* [4] and box 2 [24] (green cells in table 3), while the estimate for box 1 [24] is far from the range in less than the estimate divided by 10 (orange cell in table 3). In a similar fashion, the estimated parameters for the maximum growth rate of LAB ($\mu_{\max}^{\mathrm{LAB}}$) varied across the three datasets between 0.36 and 0.5 h^−1^, falling within the range of reported values in the literature between 0.0072 and 1.41 h^−1^ [54–59] (all parameters are within green cells in table 3). By contrast, the maximum specific growth rate of Y on Fru ($\mu_{\max}^{Y_{\mathrm{Fru}}}$) and AAB on EtOH ($\mu_{\max}^{{\mathrm{AAB}}_{\mathrm{EtOH}}}$), do not agree completely with reported values in the literature. In the first case, only the estimate obtained from box 1 conducted by Papalexandratou *et al.* [24] falls within the range of 0.01–0.166 h^−1^ [49,51–53] (green cell in table 3). In the latter case, the only estimate that does not fall within the range of 0.0106–0.25 h^−1^ [60–64] is the one obtained from the data reported by Camu *et al.* [4] (orange cell in table 3). For the maximum specific growth rate of AAB on LA ($\mu_{\max}^{{\mathrm{AAB}}_{\mathrm{LA}}}$), no values were reported in the literature for this microbial group on LA as carbon source. The estimated values for this parameter, were between 0.01 and 0.02 h^−1^.

#### 3.3.2. Substrate saturation constants

About the substrate saturation constants, here denoted by *K*, their estimated values for the three datasets agree with reported ones in the literature in one out of three instances. For these comparisons, in several occurrences a unit transformation was necessary from their original units in which they were reported to milligrams of substrate per millilitre of medium (mg(substrate) ml^−1^), and assuming that one gram of pulp is equivalent to one millilitre of medium, since our estimates are given in mg(substrate) mg(pulp)^−1^.

On the one hand, values reported in the literature for these parameters were found for the substrate saturation constants of Y on Glc ($K_{\mathrm{Glc}}^{Y}$) [46,48], Y on Fru ($K_{\mathrm{Fru}}^{Y}$) [53] and LAB on Glc ($K_{\mathrm{Glc}}^{\mathrm{LAB}}$) [57–59]. The obtained estimated values ranged from 30.01 to 35.32, 25.02 to 41.39 and 19.27 to 37.97 mg(substrate) g(pulp)^−1^, respectively. Estimated values for $K_{\mathrm{Glc}}^{Y}$ are far from the reported ranges by less than the estimate multiplied by 10 (orange cells in table 3); while for $K_{\mathrm{Fru}}^{Y}$, the estimates were further from the referenced range than the estimate multiplied by 10 (red cells in table 3). From these estimates, the ones corresponding to $K_{\mathrm{Glc}}^{\mathrm{LAB}}$ were those which fall within the reported range of 0.79–178.0 mg(Glc) ml^−1^ [57–59] (green cells in table 3).

On the other hand, no values of substrate saturation constants were reported either for AAB on EtOH ($K_{\mathrm{EtOH}}^{\mathrm{AAB}}$) or AAB on LA ($K_{\mathrm{LA}}^{\mathrm{AAB}}$). For these parameters, their estimated values ranged from 3.81 to 16.06 and 81.981 to 2509.62 mg(substrate) g(pulp)^−1^, respectively. For $K_{\mathrm{LA}}^{\mathrm{AAB}}$, this considerably higher value for the upper limit of the range was obtained for the Camu *et al.* [4] data. This inflation of values was observed for other parameters, i.e. AAB-to-EtOH yield coefficient (*Y*_EtOH |AAB_), AAB-to-LA yield coefficient (*Y*_LA |AAB_) and AAB-to-Ac from LA yield coefficient ($Y_{\mathrm{Ac}\;|\mathrm{AAB}}^{\mathrm{LA}}$), of this dataset as well.

#### 3.3.3. Mortality rate constants

For the mortality rate constants, *k*, no values were reported in the literature. Here, their estimated values for Y (*k*_Y_), LAB (*k*_LAB_) and AAB (*k*_AAB_) were in the ranges of 0.033–0.092 mg(EtOH)^−1^ h^−1^, 0.0054–0.067 mg(LA)^−1^ h^−1^ and 0.0004–0.0069 mg(Ac)^−2^ h^−1^, respectively. These estimates varied considerably between the data of Camu *et al.* [4] and Papalexandratou *et al.* [24]. In the latter, the only estimate that differed much between box 1 and box 2 was the one corresponding to *k*_Y_.

#### 3.3.4. Yield coefficients

Finally, a higher variability among the obtained parameter estimates was found for the yield coefficients, *Y*. These differences were notable between the two studies of Camu *et al.* [4] and Papalexandratou *et al.* [24], as well as the two trials (boxes 1 and 2) of the latter. In more detail, the estimated yield coefficient of Y-to-EtOH from Glc ($Y_{\mathrm{EtOH}\;|\;Y}^{\mathrm{Glc}}$) was the only one that agreed in all three datasets with those reported in the literature (green cells in table 3). Their estimated values ranged between 7.44 and 11.94 mg(EtOH)mg(Y)^−1^, falling in the referenced range of 1.39–21.49 mg(EtOH) mg(Y)^−1^ [46,49,50,68]. For the yield coefficient of LAB-to-Glc (*Y*_Glc | LAB_), the fits corresponding to the data of Camu *et al.* [4] and box 1 [24] were contained in the referenced range of 1.56–66.67 mg(Glc) mg(LAB)^−1^ [59,67] (green cells in table 3) with values of 29.23 and 20.22 mg(Glc) mg(LAB)^−1^, respectively. The remaining estimate *Y*_Glc | LAB_ for box 2 was far from the reported range in less than the estimate multiplied by 10, with a estimated mean of 3.32 mg(Glc) mg(LAB)^−1^ (orange cell in table 3).

By contrast, there are estimated yield coefficients that do not agree completely with previously reported values. On the one hand, the estimated value of 33.4 mg(Glc) mg(Y)^−1^ (green cell in table 3) for the yield coefficient of Y-to-Glc (*Y*_Glc | Y_), in the dataset of Camu *et al.* [4] only, agree with the ranges of 1.56–66.67 mg(Glc) mg(Y)^−1^ [44–46,50,51,65,66]. Their counterparts from boxes 1 and 2 reported by Papalexandratou *et al.* [24], are away from the reported range in less than the estimate multiplied by 10 (orange cells in table 3) with values of 240.93 and 119.67 mg(Glc) mg(Y)^−1^, respectively. For the yield coefficient of Y-to-Fru (*Y*_Fru | Y_), all estimated parameters are far from the referenced range of 43.48–200 mg(Fru) mg(Y)^−1^ [51,52] in less than the estimate divided by 10 (orange cells in table 3) with values between 41.11 and 244.15 mg(Fru) mg(Y)^−1^. A similar situation is observed for coefficients Y-to-EtOH from Fru ($Y_{\mathrm{EtOH}\;|\;Y}^{\mathrm{Fru}}$) and AAB-to-EtOH (*Y*_EtOH | AAB_) (orange cells in table 3), with estimated parameters between 5.927 and 11.195 mg(EtOH) mg(Y)^−1^ and a single referenced value of 5.7878 mg(EtOH) mg(Y)^−1^ [49] for $Y_{\mathrm{EtOH}\;|,textY}^{\mathrm{Fru}}$ and estimated parameters between 170.44 and 1298.070 mg(EtOH) mg(AAB)^−1^ with a referenced range of 8.06–166.67 mg(EtOH) mg(AAB)^−1^ [61,63,69] for *Y*_EtOH | AAB_.

Moreover, the estimated yield coefficients for AAB-to-LA (*Y*_LA | AAB_) do not agree in any of the data collections with the reference value of 7.94 mg(LA) mg(AAB)^−1^ [63] with values far from the reference more than 10 times the parameter for the datasets of Camu *et al.* [4] and box 1 of Papalexandratou *et al.* [24] (red cells in table 3) and one value far from the reference in less than the estimate divided by 10 for box 2 (orange cell in table 3).

For the rest of yield coefficients: LAB-to-LA (*Y*_LA | LAB_), LAB-to-Ac (*Y*_Ac | LAB_), AAB-to-Ac from EtOH ($Y_{\mathrm{Ac}\;|\;\mathrm{AAB}}^{\mathrm{EtOH}}$) and AAB-to-Ac from LA ($Y_{\mathrm{Ac}\;|\;\mathrm{AAB}}^{\mathrm{LA}}$), no values were reported in the literature. Here their estimated values were in the ranges of 2.14–10.62 mg(LA) mg(LAB)^−1^, 2.89–5.61 mg(Ac) mg(LAB)^−1^, 104.06–576.47 mg(Ac) mg(AAB)^−1^ and 385.17–1427.23 mg(Ac) mg(AAB)^−1^, respectively.

Special attention needs to be given to the values of *Y*_EtOH | AAB_, *Y*_LA | AAB_ and $Y_{\mathrm{Ac}\;|\;\mathrm{AAB}}^{\mathrm{LA}}$ for the data of Camu *et al.* [4], which showed inflated values that are not biologically plausible.

The effect of the measurement errors is discussed in electronic supplementary material, S6.

### 3.4. Statistical comparison of fermentation trials

The statistical comparison of the parameter estimates between the two fermentation trials conducted by Papalexandratou *et al.* [24] showed that significant differences exist among them (table 3), even though these were done under slightly different conditions in the same region. In this respect, the parameter estimates that showed such a significantly large difference depending on which trial they were derived from, correspond to $\mu_{\max}^{Y_{\mathrm{Glc}}}$, $\mu_{\max}^{Y_{\mathrm{Fru}}}$, $K_{\mathrm{LA}}^{\mathrm{AAB}}$, *k*_Y_, *Y*_Glc |Y_, *Y*_Glc |LAB_, *Y*_EtOH |AAB_ and *Y*_LA |AAB_. In the comparison of all these parameters, the computed absolute value of the standardized mean difference (effect size *d*) was greater than the threshold of 1.2 suggested by Sawilowsky [43]. Such a result leads to hypothesize that minor changes in both, methodologies and regions, affect the parameters of the model as it will be addressed in the discussion section.

## 4. Discussion

### 4.1. Model fitting

As shown in the aforementioned results, our current model for cocoa bean fermentation is capable of reproducing each of the datasets with high accuracy. This means that the mechanistic assumptions made here are in accordance with the available biological knowledge to a considerably good degree, as it has been reflected in the conducted simulations. In this sense, our model represents a mechanistic approach which allows for a deep understanding of the transient responses of the process in a dynamic way, as opposed to current metabolic flux analyses [15,16] that assume steady-state metabolic conditions. Moreover, it represents a fully working kinetic model as opposed to a previous attempt which is capable of simulating metabolites and products time-courses only [17].

However, a detailed analysis of the resulting fits provides insight into the validity of some of the regulatory assumptions underlying the model, the relevance of additional effects not included in the present version of the model, as well as differences between the experimental set-ups behind the datasets.

### 4.2. Regulatory assumptions

By analysing the parameter estimates obtained here, important features of the model can be explored for its enhancement in future iterations. A Bayesian framework for parameter estimation, as used here, provides a scheme to investigate their uncertainty and determine their possible uniqueness. Therefore, it serves as a descriptive source for deriving the plausibility of the regulatory assumptions of the model.

In this sense, one of the particularities of the fitted model is the presence of strongly elevated estimates for the data of Camu *et al.* [4]. Specifically, the parameters showing such values were: (i) the substrate saturation constant $K_{\mathrm{LA}}^{\mathrm{AAB}}$ and (ii) the yield coefficients *Y*_EtOH |AAB_, *Y*_LA |AAB_ and $Y_{\mathrm{Ac}\;|\mathrm{AAB}}^{\mathrm{LA}}$. Looking at the standard deviations (table 3, electronic supplementary material, S4, figures S9–S11) of their corresponding posterior distributions, it can be noted that there is a huge uncertainty in their values (large errors). These uncertainties reveal that the parameters cannot be uniquely estimated from these particular data, suggesting a practical non-identifiability of the parameters with the data reported by Camu *et al.* [4]. The reason for this characteristic can be threefold. Firstly, noise in the experimental data prevents a unique determination of the model’s parameters because of an insufficient signal-to-noise response [70]. Secondly, the data may be incongruent particularly with the model mechanisms of growth of AAB on LA and the interactions AAB–EtOH. Finally, the estimated parameters might be correlated.

In our opinion, all elevated parameters related to the growth of AAB on LA, i.e. $K_{\mathrm{LA}}^{\mathrm{AAB}}$, *Y*_LA | AAB_ and $Y_{\mathrm{Ac}\;|\;\mathrm{AAB}}^{\mathrm{LA}}$, can be a result of noise in experimental data. Thus, as revealed by visual inspection of figure 3*d* where the real data does not reflect an accentuated decrease in the concentration of LA as opposed to the data reported by Papalexandratou *et al.* [24] (figures 4*d* and 5*d*), where such decrease exists after 72 h of the fermentation process. For the remaining elevated parameter which is related to the consumption of EtOH by AAB (*Y*_EtOH |AAB_), a straight interpretation of its value of ≈1300 mg(Glc) mg(Y)^−1^ would imply that 1300 mg of EtOH are consumed by 1 mg of AAB; or in other words, that for generating 1 mg of AAB, 1300 mg of EtOH is required. Obviously, a yield coefficient of this order of magnitude is biologically implausible and for this reason it could be argued that the proposed model is not entirely capturing all the inherent mechanisms of the AAB–EtOH interaction in the fermentation process. A possible explanation of this specific inflated parameter value, is that *Y*_EtOH |AAB_ is not only capturing the consumption of EtOH by AAB, but also possible physical processes such as evaporation of this metabolite. Temperature data, so far not implemented in the model, gave the reason for this hypothesis. More precisely, the data of Camu *et al.* [4] shows higher temperatures of the fermentation mass much earlier in the process compared to the data reported by Papalexandratou *et al.* [24]. In the first case, a temperature above 35°C was reached right after 30 h and its maximum of ≈45°C at 70 h of the fermentation process. In the latter, similar temperatures were reached at 40 and 80 h of the process. This disparity might explain why the estimated parameter values for *Y*_EtOH | AAB_ in the Papalexandratou *et al.* [24] data collection are between 3 to 5 times less compared to the value obtained for the data of Camu *et al.* [4]. Finally, correlation between the estimated parameters might be playing an important role in the non-identifiability of these inflated parameters. In other words, interdependencies between different sets of parameters limit the MCMC sampler to freely explore the solution space. In that sense, from a simple correlation analysis (see electronic supplementary material, S7), we did not find remarkable patterns among the posterior probabilities of the parameter estimates, not even in the inflated ones.

According to these hypotheses, further iterations of the model should include additional physical effects, especially temperature. Moreover, pH conditions during the conduction of the fermentation should be also taken into account, as well as a non-dimensionalization of the model to identify correlated estimates and reduce their number.

### 4.3. Parameter conformance to values in the literature

The parameter ranges indicated as *reported values* in table 3 are in many cases referring to different experimental conditions and/or a specific microorganismal strain and might therefore not be directly comparable to the biological situation discussed here. We resorted to these values, whenever we failed to identify parameter values directly applicable to cocoa bean fermentation, in order to at least provide an order-of-magnitude estimate.

Hence, among the different estimated parameters, few did not agree with previously reported values in the literature as pointed out in §3.3. For the substrate saturation constants $K_{\mathrm{Glc}}^{Y}$ and $K_{\mathrm{Fru}}^{Y}$, such a difference can be explained by the fact that the reported values correspond to the growth of Y under aerobic conditions, as opposed to the anaerobic earlier stage of cocoa bean fermentation where Y’s growth takes place. Consequently, higher values for these parameters as reported here, reflect a slow uptake rate of Glc and Fru by Y under anaerobic conditions. Finally, from a general point of view, the various discrepancies between the obtained parameter estimates for the growth rates and yield coefficients with their values reported in the literature for single species confirms the high growth rates and yields coefficients that mixtures of microorganisms might show in fermentation processes [71].

### 4.4. Comparison of parameter estimates

As mentioned in §2.1, the study performed by Papalexandratou *et al.* [24], involved two fermentation trials conducted in two cocoa-producing farms in Brazil belonging to the same region. These trials denoted as ‘box 1’ and ‘box 2’, differed between each other in minor aspects. After we identified eight significantly different estimates between these trials (table 3), in the following paragraphs, three main differences were taken into account in order to formulate hypotheses on how the parameter estimates are affected when applying the same fermentation method, i.e. wooden boxes, under similar environmental conditions in distinct fermentations. The three main differences between the trials are: (i) initial concentration of microorganisms, (ii) concentration ratios of initial substrates, and (iii) evaporation rates.

#### 4.4.1. Initial concentration of microorganisms

The initial concentrations of microorganisms between boxes 1 and 2 did not differ much, with the exception of Y. For Y, the initial concentrations in boxes 1 and 2 were equal to 0.18 and 0.005 mg g^−1^, respectively. This difference as well as the microbial diversity that has been seen along different fermentation trials [2] determined that the estimates of these growth rates differ between each fermentation trial; with higher values of the growth rates $\mu_{\max}^{Y_{\mathrm{Glc}}}$ and $\mu_{\max}^{Y_{\mathrm{Fru}}}$ for box 2 than for box 1. A similar effect is evident in the yield coefficient related to the growth of Y on Glc (*Y*_Glc |Y_), where box 1 showed a higher value than the one obtained for box 2 as a consequence of the higher initial amount of Y in box 1. In other words, a higher initial concentration of Y determines the estimation of lower maximum specific growth rates as well as higher estimates for the yield coefficient of Y on Glc. This can be explained that given a high initial microbial population, it needs a lower cell division rate to reach the maximum described by the observed data and an increased rate of uptake of its substrate.

#### 4.4.2. Concentration ratios of initial substrates

Worthy of attention, was the difference in the initial concentrations of the main substrates Glc and Fru which might play an important role in the growth of Y and LAB. For box 1, the initial concentrations of Glc and Fru are 55.482 and 49.669 mg g^−1^, respectively; while for box 2, these are 42.936 and 67.249 mg g^−1^, respectively. This ratio would explain the significant difference in the yield coefficient of the growth of LAB on Glc (*Y*_Glc |LAB_) between boxes 1 and 2. In box 2, this yield coefficient was estimated significantly lower than for box 1 which leads to the hypothesis that this phenomena might be the result of a growing population of Y restraining the access to Glc to the LAB microbial group. This final hypothesis coincides with our assumption of resource-type competition between Y and LAB [34,35], which determined a less successful *Y*_Glc | LAB_ for LAB under a lower initial concentration of its main substrate Glc in box 2.

#### 4.4.3. Evaporation rates

Among the subtle differences between boxes 1 and 2 reported by Papalexandratou *et al.* [24], the final one to be considered is the possible uneven evaporation rates between them. With this in mind, it is not deceitful to expect a higher evaporation rate of volatile metabolites in a fermenting mass protected only by a metal roof, as reported for box 1, than in a fermenting mass held within a fermentary room, as reported for box 2. In this respect, the higher estimated yield coefficient of growth of AAB on EtOH (*Y*_EtOH |AAB_) in box 1 than in box 2, might be explained for a possibly greater evaporation rate of EtOH in box 1, similar to the possible explanation of its inflated counterpart determined for the Camu *et al.* [4] dataset. A similarly higher evaporation rate for LA and Ac in box 1 could be the explanation for the differences among the remaining parameter estimates, i.e. the Contois substrate saturation constant for the growth of AAB on LA ($K_{\mathrm{LA}}^{\mathrm{AAB}}$), the yield coefficient of consumption of LA by AAB (*Y*_LA |AAB_) as well as the large variances of the yield coefficients of the production of Ac from EtOH and LA by AAB ($Y_{\mathrm{Ac}\;|\;\mathrm{AAB}}^{\mathrm{EtOH}}$ and $Y_{\mathrm{Ac}\;|\;\mathrm{AAB}}^{\mathrm{LA}}$, respectively). This hypothesis can be also extended to the lower value in the mortality rate term of Y (*k*_Y_) observed in box 1 than in box 2, where EtOH loses its effect on decreasing Y’s population due to a higher evaporation. In other words, the mortality rate of Y might be lowered in the presence of an increasing evaporation rate of EtOH in the fermenting mass.

## 5. Conclusion

The model presented here is a first biochemically plausible, ODE-based kinetic model of cocoa bean fermentation capable of reproducing the known sequential activation of microbial communities and capable of fitting available experimental data to an acceptable degree. However, it is necessarily a simplification of the diverse biological processes involved in cocoa bean fermentation. The remaining discrepancies between model prediction and experimental data, as well as those parameter values outside the biologically plausible ranges, point to the fact that relevant aspects of the processes have not been taken into account.

Based on the model features, we can hypothesize that the following regulatory mechanisms might exist: (i) resource-type competition between Y and LAB, (ii) microbial death is determined to a good degree by direct action of fermentation products upon their respective producing microorganisms and (iii) chemical and physical factors intervene in the decrement of volatile products, i.e. EtOH and LA, rather than microbial activities only.

This mathematical model allows relating observed microbial population sizes and concentrations of the five chemical compounds considered here, i.e. Glc, Fru, EtOH, LA and Ac, during the time course of fermentation with growth rates, mortality rates, substrate saturation constants and yield coefficients as intrinsic systemic parameters.

Additionally, the capability of the model to ‘reverse-engineer’ differences from the observed time courses of two trials conducted in the same region under the same methodology showed how these systemic parameters might be affected by minor changes between one and other fermentation trial. The cocoa and chocolate markets require a steady flow of high-quality raw material resulting from diverse types of fermentation. Although fermentation practices will remain locally determined, model-based recommendations to farmers on practices and use of specific starter cultures might help increase the quality of cocoa bean raw material prior to shipment. This will ultimately increase the sustainability of cocoa bean supply.

Subsequent versions of the model should include additional chemical and physical effects, such as temperature and pH dependence of kinetic parameters, the spatial heterogeneity of a fermentation pile, impact of additional (commonly occurring) microorganisms, as well as a further compartmentalization including the inner bean and the incorporation of sucrose as an additional carbon source, serving also as a (time-delayed) source of glucose and fructose.

Besides the extension by further chemical and physical effects, eventually such a kinetic model needs to be interfaced with the more microscopic, metabolic perspective put forward in other studies [15,16]. Should high-quality genome-scale metabolic models be available, flux balance analysis [72] may provide a suitable theoretical framework for such an approach on three levels: (i) A metabolic pathway analysis of synergies and competitions (e.g. using the methodology from [73]) may point to additional modes of interaction among the species involved. (ii) A detailed exploration of the emerging pattern of chemical compounds as a function of the fermentation time course may become feasible by incorporating the biochemical interactions of the cocoa bean and the microorganisms. (iii) The relevance of a larger diversity in microorganisms (e.g. different yeast strains) can be assessed. With the availability of genome-scale metabolic models currently developing rapidly [74], we expect this avenue of research to become feasible in the very near future.

The recent finding [75] about the metabolic interplay of yeast and LAB is an example of the richness of this metabolic foundation underlying the dynamics leading to the successful fermentation of a cocoa bean.

## Supplementary Material

A mathematical model of cocoa bean fermentation: Supplementary Material

## Data accessibility

The datasets and codes supporting this article are deposited at Dryad Digital Repository: https://dx.doi.org/10.5061/dryad.321d33v [76].
