# Supplementary material for: A mathematical model of cocoa bean fermentation
Source: R Soc Open Sci. 2018 Oct 17;5(10):180964. doi: 10.1098/rsos.180964 (PMC6227950; doi:10.1098/rsos.180964)
Supplement: A mathematical model of cocoa bean fermentation: Supplementary Material [file rsos180964supp1.pdf]

Electronic Supplementary Material for:

A mathematical model of cocoa bean fermentation

Journal of Royal Society – Open Science

Mauricio Moreno-Zambrano[1], Sergio Grimbs[1], Matthias S. Ullrich[1], and Marc-Thorsten Hütt[1]

[1]Department of Life Sciences and Chemistry, Jacobs University Bremen, Campus Ring 1, 28759 Bremen, Germany

NOTE: In the following, references to tables, equations or figures in the Main Manuscript are labelled with the acronym MM. For example, table 1, equation (2.1) and figure 1 of the Main Manuscript are referred as table 1.MM, equation (2.1.MM) and figure 1.MM respectively.

Supplementary Material S1: Geometric derivation of conversion factors between CFU to mass

According to the Bergey's Manual of Systematic Bacteriology [1, 2], the diameter of *Lactobacillus plantarum* (representative species of lactic acid bacteria in cocoa bean fermentation) is between 0.9 to 1.2 µm and its length between 3 to 8 µm. For the case of the *Acetobacter* genus, its usual diameter is between 0.6 to 0.9 µm and its length is between 1.0 to 4.0 µm.

Given the volume of a spherocylinder as

$$V = \pi r^2 \left(\frac{4}{3} r + a \right) ,$$

a midpoint from the above ranges can be computed in order to determine a theoretical volume for a single cell, where a is equal to the length minus two times the radius (r). Thus, for lactic acid bacteria (LAB) the volume is equal to 4.46 μm^3 and for acetic acid bacteria (AAB) it is ≈ 1 μm^3. Therefore; using as reference the dry weight of a single cell of *E. coli* reported by Neidhardt & Umbarger [3] of 2.8×10^{-10} mg and its volume of 1 μm^3 [4], the conversion factor between CFU to dry biomass of LAB and AAB were determined as 1.25 and 0.28 $\mathrm{pg\,CFU^{-1}}$ respectively.

Supplementary Material S2: Model's iterations

Baseline model (M1)
In a first instance, a baseline model (M1) was proposed in an oversimplified manner. For M1, the main substrates (glucose (Glc) and fructose (Fru)) were combined in a single state variable named 'monosaccharides' (M). Furthermore, it comprehends simple coupled interactions among the state variables reduced to single uptake of substrates and production of metabolites. A network diagram of M1 is shown in Figure S1.

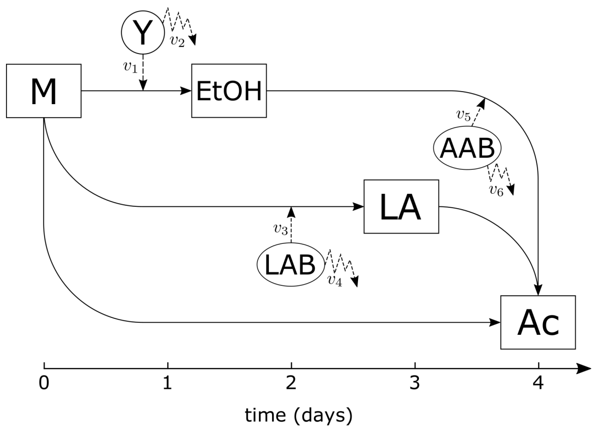

Figure S1: Network diagram of the cocoa bean fermentation baseline model (M1). Microbial groups: yeast (Y), lactic acid bacteria (LAB) and acetic acid bacteria (AAB) are represented as circles. Metabolites: monosaccharides (M), ethanol (EtOH), lactic acid (LA) and acetic acid (Ac) are represented as squares. The growth rates of yeast on monosaccharides (v_1), of lactic acid bacteria on monosaccharides (v_3), and of acetic acid bacteria on ethanol (v_5) are represented as straight dashed arrows. The mortality rates of yeast (v_2), lactic acid bacteria (v_4), and acetic acid bacteria (v_6) are represented as zigzag dashed arrows. Solid straight arrows show the direction in which the conversion of metabolites occurs.

The system of ODE's for M1 comprises Monod equations for the growth rates and first-order reaction kinetics for the mortality rates, as shown in Table S1.

Table S1: Growth and mortality rate equations for the cocoa bean fermentation baseline model (M1). Microbial groups are represented as yeast (Y), lactic acid bacteria (LAB) and acetic acid bacteria (AAB). Substrates are represented as monosaccharides (M) and ethanol (EtOH). Biomass and concentration of substrates, both are shown within square brackets [ ]. Maximum specific growth rates μ_{max} are shown of the form μ^{i}_{max}, where i can be either Y, LAB and AAB. Substrate saturation constants for the growth of Y, LAB and AAB are shown of the form K^{j}_{m}, where j can be either Y or LAB and m can be either M or EtOH. Constant mortality rates are shown of the form k_i, where i can be either Y, LAB or AAB.

Growth rate equation	Mortality rate equation
$v_1 = \dfrac{\mu^{\text{Y}}_{\text{max}}\ [\text{M}]}{[\text{M}] + K^{\text{Y}}_{\text{M}}}\ [\text{Y}]$	$v_2 = k_{\text{Y}}\ [\text{Y}]$
$v_3 = \dfrac{\mu^{\text{LAB}}_{\text{max}}\ [\text{M}]}{[\text{M}] + K^{\text{LAB}}_{\text{M}}}\ [\text{LAB}]$	$v_4 = k_{\text{LAB}}\ [\text{LAB}]$
$v_5 = \dfrac{\mu^{\text{AAB}}_{\text{max}}\ [\text{EtOH}]}{[\text{EtOH}] + K^{\text{AAB}}_{\text{EtOH}}}\ [\text{AAB}]$	$v_6 = k_{\text{AAB}}\ [\text{AAB}]$

In this way, M1 is constructed as a system of ODE's with seven state variables with six yield

coefficients, as expressed in Eqs. (1) to (7).

$$\frac{d\,[\mathrm{M}]}{dt} = -Y_{\mathrm{M|Y}}\, v_1 - Y_{\mathrm{M|LAB}}\, v_3 \tag{1}$$

$$\frac{d\,[\mathrm{EtOH}]}{dt} = Y_{\mathrm{EtOH|Y}}\, v_1 - Y_{\mathrm{EtOH|AAB}}\, v_5 \tag{2}$$

$$\frac{d\,[\mathrm{LA}]}{dt} = Y_{\mathrm{LA|LAB}}\, v_3 \tag{3}$$

$$\frac{d\,[\mathrm{Ac}]}{dt} = Y_{\mathrm{Ac|AAB}}\, v_5 \tag{4}$$

$$\frac{d\,[\mathrm{Y}]}{dt} = v_1 - v_2 \tag{5}$$

$$\frac{d\,[\mathrm{LAB}]}{dt} = v_3 - v_4 \tag{6}$$

$$\frac{d\,[\mathrm{AAB}]}{dt} = v_5 - v_6 \tag{7}$$

Model's iterations

For the formulation of further model iterations, the 'monosaccharides' state variable in M1 was considered by their separate components (Glc and Fru) and different possible mechanisms of growth as well as mortality rates were taken into account.

Among the growth rates, it were considered the Monod [5] and Contois [6] equations as possible candidate terms. Specifically, the Contois [6] equation was taken into account as candidate for the growth rate of acetic acid bacteria (AAB) on lactic acid (LA) (equation v_5, in table 1.MM). This assumption relies on the following premise: given that few species of AAB are capable of catabolize lactic acid [7, 8], it can be argued that the growth rate of these species is a function of their population size. In that manner, the cell growth of AAB on LA is reduced along the population size of these particular AAB species increases during the fermentation process.

For the mortality rates, linear and non-linear terms of mortality rates were considered. On the one hand, linear terms were evaluated as commonly used first-order reaction terms (mortality constant by concentration of microorganisms at a given time). On the other hand, non-linear mortality rates were defined by the multiplication of first-order reaction terms with the correspondent products that each microbial group produces in the process (Chick-Watson mortality law [9]).

Once considering previously reported interactions between the state variables of the model [10–14], the use of two different mortality rate types determines two network diagram representations in which, one does not contain effect of products upon their producer microbial populations (Figure S2 (a)) and the one corresponding to the network diagram presented in figure 2.MM (Figure S2 (b)).

After several preliminary model iterations, it were found (results not shown) that non-linear mortality rate terms which showed better fits with respect to the experimental data of Camu *et al.* [11] and Papalexandratou *et al.* [15] (visually inspected) were those presented in table 1.MM (v_6, v_7 and v_8). From these considerations, three model iterations were conducted using the same Bayesian framework depicted in the 'Material and methods' section of the manuscript and evaluated in terms of the leave-one-out cross-validation (LOO) and the widely applicable information criterion (WAIC) for Bayesian model selection[16, 17]. A sum of the obtained Root Mean Square Error (RMSE) per Markov chain

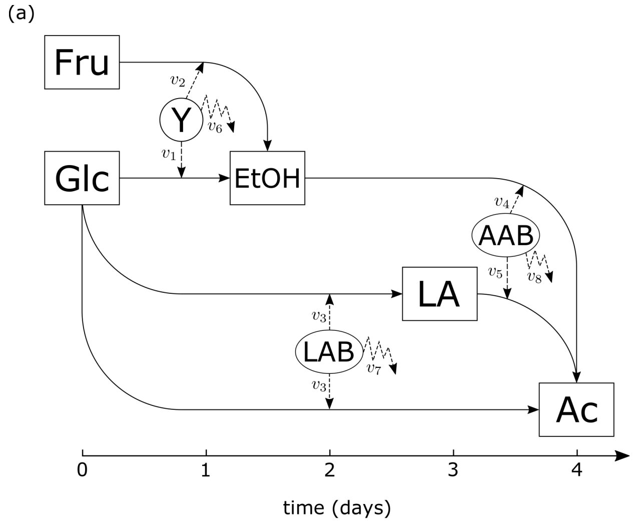

Figure S2: Network diagram of the cocoa bean fermentation model. (a) Considering linear mortality rates. (b) Considering non-linear mortality rates (red dashed arrows). Microbial groups: yeast (Y), lactic acid bacteria (LAB) and acetic acid bacteria (AAB) are represented as circles. Metabolites: Glucose (Glc), fructose (Fru), ethanol (EtOH), lactic acid (LA) and acetic acid (Ac) are represented as squares. The growth rates of yeast on glucose (v_1) and fructose (v_2), of lactic acid bacteria (v_3), and of acetic acid bacteria on ethanol (v_4) and acetic acid (v_5) are represented as straight dashed arrows. The mortality rates of yeast (v_6), lactic acid bacteria (v_7) and acetic acid bacteria (v_8) are represented as zigzag dashed arrows. Solid straight arrows show the direction in which the conversion of metabolites occur.

Monte Carlo (MCMC) for each of the time-series in each model iteration was also computed.

The characteristics of each model, including the baseline model, is shown in Table S2.

Table S2: Summary of the model's iterations. Check-marks and cross-marks indicates whether the model includes the use of multiple substrates (Glc and Fru), product toxicity interactions (non-linear mortality rates) and population size effect for the consumption of lactic acid (LA) in the form of a Contois term for the growth of acetic acid bacteria (AAB) on LA.

Model	Multiple substrate	Product toxicity	Population size effect for LA consumption
M1	✗	✗	✗
M2	✓	✗	✗
M3	✓	✓	✗
M4	✓	✓	✓

For the RMSE for a t time-series is defined as:

$$\text{RMSE}_t = \sqrt{\frac{1}{N}\sum_{n=1}^{N}(\hat{x}_n - x_n)^2}\ , \tag{8}$$

where $\hat{x}_n$ represents the mean of the posterior distribution obtained for the x_n observed data at time point n, and N is the total number of observations for a t time-series.

Therefore, a total RMSE for the model (RMSE_T) is defined by the sum of the obtained RMSE_t

for each time-series defined in Eq. (8):

$$\text{RMSE}_T = \sum_{t=1}^{T} \text{RMSE}_t \ . \tag{9}$$

For comparison purposes of M1 with the model's iterations (M2, M3 and M4), Eq. (9) is reformulated as:

$$\text{RMSE}_T = \frac{1}{S} \sum_{t=1}^{T} \text{RMSE}_t \ , \tag{10}$$

where S is the number of state variables in the model.

Model simulation results

It was obtained that for the three datasets, the estimated values of LOO and WAIC (Figure S3) for M1 have worse performances than for the model's iterations. M3 and M4 showed better results for all datasets, with the exception of data corresponding to Box 1 (Figure S3, panel (b)) where M2 showed a better performance.

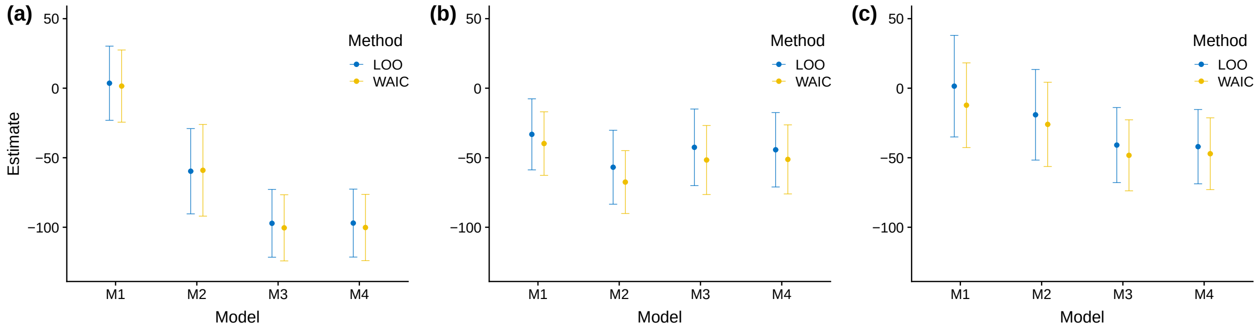

Figure S3: Leave-one-out cross-validation (LOO) and widely applicable information criterion (WAIC) for models M1 (baseline), M2, M4 and M5. Dots represents the estimates for LOO and WAIC, lines represent their standard error. (a) Camu *et al.* [11] dataset, (b) Box 1 of Papalexandratou *et al.* [15] and (c) Box 2 of Papalexandratou *et al.* [15].

Similar results were obtained for the RMSE_T (Figure S4).

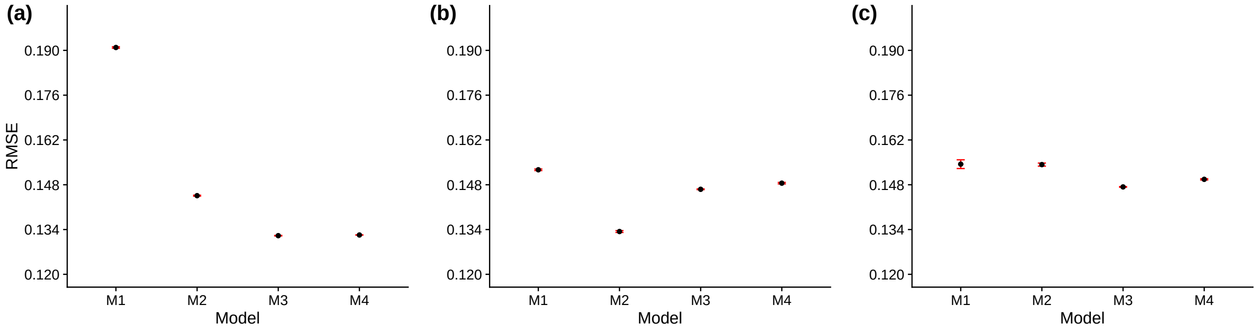

Figure S4: Total Root Mean Square Error (RMSE_T) for models M1 (baseline), M2, M4 and M5. Black dots represents the mean of the RMSE_T along the 4 MCMC, red lines represent their standard deviation. (a) Camu *et al.* [11] dataset, (b) Box 1 of Papalexandratou *et al.* [15] and (c) Box 2 of Papalexandratou *et al.* [15].

However, M4 was selected as the most plausible model iteration among the three datasets because M3 showed a bimodal posterior distribution for the maximum specific growth rate of AAB on EtOH ($\mu_{\max}^{\text{AAB}_{\text{EtOH}}}$) with the data from Camu *et al.* [11] as shown in Figure S5.

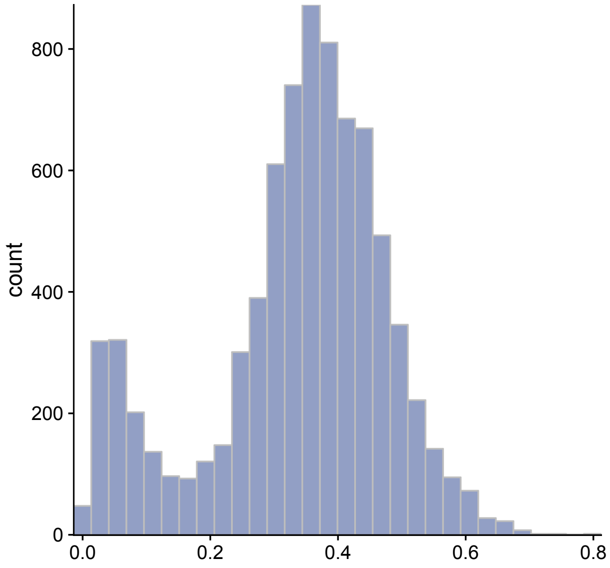

Figure S5: Posterior distribution of the maximum specific growth rate of AAB on EtOH ($\mu_{\max}^{\mathrm{AAB}_{\mathrm{EtOH}}}$) of model M3 with the data from Camu *et al.* [11].

Supplementary Material S3: Analytical determination of conversion factors for parameter estimates

Consider the kinetics of any x state variable in the proposed model, its concentration $[x]$ in a certain time and the maximum concentration $[x]_{max}$ within the given time interval in which the fermentation process took place.

Let's x' represent a transformation of the estate variable x expressed as

$$x' = \frac{[x]}{[x]_{max}}, \tag{11}$$

then, the time derivative of x' will be given by

$$\frac{dx'}{dt} = \frac{1}{[x]_{max}} \frac{d\,[x]}{dt}, \tag{12}$$

where $[x]_{max}$ is either the maximum concentration of glucose, fructose, ethanol, lactic acid, acetic acid, yeast, lactic acid bacteria or acetic acid bacteria, depending on which ODE of the proposed model is expressed in the form of Eq. (12).

Generalizing, by expressing all ODEs of the model in the form of Eq. (12), one gets a system of equations as

$$\frac{1}{[\mathrm{Glc}]_{max}} \frac{d\,[\mathrm{Glc}]}{dt} = -Y_1 \frac{\mu_1 \frac{[\mathrm{Glc}]}{[\mathrm{Glc}]_{max}}}{\frac{[\mathrm{Glc}]}{[\mathrm{Glc}]_{max}} + Ks_1} \frac{[\mathrm{Y}]}{[\mathrm{Y}]_{max}} - Y_2 \frac{\mu_3 \frac{[\mathrm{Glc}]}{[\mathrm{Glc}]_{max}}}{\frac{[\mathrm{Glc}]}{[\mathrm{Glc}]_{max}} + Ks_3} \frac{[\mathrm{LAB}]}{[\mathrm{LAB}]_{max}}, \tag{13}$$

$$\frac{1}{[\mathrm{Fru}]_{max}} \frac{d\,[\mathrm{Fru}]}{dt} = -Y_3 \frac{\mu_2 \frac{[\mathrm{Fru}]}{[\mathrm{Fru}]_{max}}}{\frac{[\mathrm{Fru}]}{[\mathrm{Fru}]_{max}} + Ks_2} \frac{[\mathrm{Y}]}{[\mathrm{Y}]_{max}}, \tag{14}$$

$$\begin{aligned}\frac{1}{[\mathrm{EtOH}]_{max}} \frac{d\,[\mathrm{EtOH}]}{dt} = {} & Y_4 \frac{\mu_1 \frac{[\mathrm{Glc}]}{[\mathrm{Glc}]_{max}}}{\frac{[\mathrm{Glc}]}{[\mathrm{Glc}]_{max}} + Ks_1} \frac{[\mathrm{Y}]}{[\mathrm{Y}]_{max}} + Y_5 \frac{\mu_2 \frac{[\mathrm{Fru}]}{[\mathrm{Fru}]_{max}}}{\frac{[\mathrm{Fru}]}{[\mathrm{Fru}]_{max}} + Ks_2} \frac{[\mathrm{Y}]}{[\mathrm{Y}]_{max}} \\ & - Y_6 \frac{\mu_4 \frac{[\mathrm{EtOH}]}{[\mathrm{EtOH}]_{max}}}{\frac{[\mathrm{EtOH}]}{[\mathrm{EtOH}]_{max}} + Ks_4} \frac{[\mathrm{AAB}]}{[\mathrm{AAB}]_{max}},\end{aligned} \tag{15}$$

$$\frac{1}{[\mathrm{LA}]_{max}} \frac{d\,[\mathrm{LA}]}{dt} = Y_7 \frac{\mu_3 \frac{[\mathrm{Glc}]}{[\mathrm{Glc}]_{max}}}{\frac{[\mathrm{Glc}]}{[\mathrm{Glc}]_{max}} + Ks_3} \frac{[\mathrm{LAB}]}{[\mathrm{LAB}]_{max}} - Y_8 \frac{\mu_5 \frac{[\mathrm{LA}]}{[\mathrm{LA}]_{max}}}{\frac{[\mathrm{LA}]}{[\mathrm{LA}]_{max}} + Ks_5 \frac{[\mathrm{AAB}]}{[\mathrm{AAB}]_{max}}} \frac{[\mathrm{AAB}]}{[\mathrm{AAB}]_{max}}, \tag{16}$$

$$\begin{aligned}\frac{1}{[\mathrm{Ac}]_{max}} \frac{d\,[\mathrm{Ac}]}{dt} = {} & Y_9 \frac{\mu_3 \frac{[\mathrm{Glc}]}{[\mathrm{Glc}]_{max}}}{\frac{[\mathrm{Glc}]}{[\mathrm{Glc}]_{max}} + Ks_3} \frac{[\mathrm{LAB}]}{[\mathrm{LAB}]_{max}} + Y_{10} \frac{\mu_4 \frac{[\mathrm{EtOH}]}{[\mathrm{EtOH}]_{max}}}{\frac{[\mathrm{EtOH}]}{[\mathrm{EtOH}]_{max}} + Ks_4} \frac{[\mathrm{AAB}]}{[\mathrm{AAB}]_{max}} \\ & + Y_{11} \frac{\mu_5 \frac{[\mathrm{LA}]}{[\mathrm{LA}]_{max}}}{\frac{[\mathrm{LA}]}{[\mathrm{LA}]_{max}} + Ks_5 \frac{[\mathrm{AAB}]}{[\mathrm{AAB}]_{max}}} \frac{[\mathrm{AAB}]}{[\mathrm{AAB}]_{max}},\end{aligned} \tag{17}$$

$$\frac{1}{[\mathrm{Y}]_{max}}\frac{d\,[\mathrm{Y}]}{dt}=\frac{\mu_1\,\frac{[\mathrm{Glc}]}{[\mathrm{Glc}]_{max}}}{\frac{[\mathrm{Glc}]}{[\mathrm{Glc}]_{max}}+Ks_1}\,\frac{[\mathrm{Y}]}{[\mathrm{Y}]_{max}}+\frac{\mu_2\,\frac{[\mathrm{Fru}]}{[\mathrm{Fru}]_{max}}}{\frac{[\mathrm{Fru}]}{[\mathrm{Fru}]_{max}}+Ks_2}\,\frac{[\mathrm{Y}]}{[\mathrm{Y}]_{max}}-k_1\,\frac{[\mathrm{Y}]}{[\mathrm{Y}]_{max}}\,\frac{[\mathrm{EtOH}]}{[\mathrm{EtOH}]_{max}},\tag{18}$$

$$\frac{1}{[\mathrm{LAB}]_{max}}\frac{d\,[\mathrm{LAB}]}{dt}=\frac{\mu_3\,\frac{[\mathrm{Glc}]}{[\mathrm{Glc}]_{max}}}{\frac{[\mathrm{Glc}]}{[\mathrm{Glc}]_{max}}+Ks_3}\,\frac{[\mathrm{LAB}]}{[\mathrm{LAB}]_{max}}-k_2\,\frac{[\mathrm{LAB}]}{[\mathrm{LAB}]_{max}}\,\frac{[\mathrm{LA}]}{[\mathrm{LA}]_{max}},\tag{19}$$

$$\begin{aligned}\frac{1}{[\mathrm{AAB}]_{max}}\frac{d\,[\mathrm{AAB}]}{dt}=&\frac{\mu_4\,\frac{[\mathrm{EtOH}]}{[\mathrm{EtOH}]_{max}}}{\frac{[\mathrm{EtOH}]}{[\mathrm{EtOH}]_{max}}+Ks_4}\,\frac{[\mathrm{AAB}]}{[\mathrm{AAB}]_{max}}+\frac{\mu_5\,\frac{[\mathrm{LA}]}{[\mathrm{LA}]_{max}}}{\frac{[\mathrm{LA}]}{[\mathrm{LA}]_{max}}+Ks_5\frac{[\mathrm{AAB}]}{[\mathrm{AAB}]_{max}}}\,\frac{[\mathrm{AAB}]}{[\mathrm{AAB}]_{max}}\\&-k_3\,\frac{[\mathrm{AAB}]}{[\mathrm{AAB}]_{max}}\,\frac{[\mathrm{Ac}]^2}{[\mathrm{Ac}]^2_{max}},\end{aligned}\tag{20}$$

which constitutes a scaled version of the proposed model, where μ_i, Ks_i, Y_i and k_i are the solutions of the ODEs upon their correspondent scaled time series (according to Eq. (11)) for specific maximum growth rates, saturation constants, yield coefficients and mortality rates respectively.

Once the parameters of Eqs. (13) to (20) are estimated as result of the Bayesian optimization routine, obtaining conversion factors to their real units is accomplished by properly working out the equations in such a way that the maximum values of each time series are multiplied by the parameters' scaled solutions.

For instance, Eq. (13) can be simplified to:

$$\frac{d\,[\mathrm{Glc}]}{dt}=-Y_1\,\frac{[\mathrm{Glc}]_{max}}{[\mathrm{Y}]_{max}}\,\frac{\mu_1\,[\mathrm{Glc}]}{[\mathrm{Glc}]+Ks_1\,[\mathrm{Glc}]_{max}}\,[\mathrm{Y}]-Y_2\,\frac{[\mathrm{Glc}]_{max}}{[\mathrm{LAB}]_{max}}\,\frac{\mu_3\,[\mathrm{Glc}]}{[\mathrm{Glc}]+Ks_3\,[\mathrm{Glc}]_{max}}\,[\mathrm{LAB}]\,,\tag{21}$$

where the terms μ_1, μ_3, $Ks_1\,[\mathrm{Glc}]_{max}$, $Ks_2\,[\mathrm{Glc}]_{max}$, $Y_1\,\frac{[\mathrm{Glc}]_{max}}{[\mathrm{Y}]_{max}}$ and $Y_2\,\frac{[\mathrm{Glc}]_{max}}{[\mathrm{LAB}]_{max}}$ are equivalent to $\mu_{\mathrm{max}}^{\mathrm{Y_{Glc}}}$, $\mu_{\mathrm{max}}^{\mathrm{LAB}}$, $K_{\mathrm{Glc}}^{\mathrm{Y}}$, $K_{\mathrm{Glc}}^{\mathrm{LAB}}$, $Y_{\mathrm{Glc|Y}}$ and $Y_{\mathrm{Glc|LAB}}$ respectively and the left hand side of the equation corresponds to the state variable with no scaling.

Thus, similarly to Eq. (21), conversion factors between the scaled parameters to its real units can be determined as

$$\mu_{\mathrm{max}}^{\mathrm{Y_{Glc}}}=\mu_1$$
$$\mu_{\mathrm{max}}^{\mathrm{Y_{Fru}}}=\mu_2$$
$$\mu_{\mathrm{max}}^{\mathrm{LAB}}=\mu_3$$
$$\mu_{\mathrm{max}}^{\mathrm{AAB_{EtOH}}}=\mu_4$$
$$\mu_{\mathrm{max}}^{\mathrm{AAB_{LA}}}=\mu_5$$
$$K_{\mathrm{Glc}}^{\mathrm{Y}}=Ks_1\,[\mathrm{Glc}]_{\mathrm{max}}$$
$$K_{\mathrm{Fru}}^{\mathrm{Y}}=Ks_2\,[\mathrm{Fru}]_{\mathrm{max}}$$

$$K_{\mathrm{Glc}}^{\mathrm{LAB}}=Ks_3\,[\mathrm{Glc}]_{\mathrm{max}}$$
$$K_{\mathrm{EtOH}}^{\mathrm{AAB}}=Ks_4\,[\mathrm{EtOH}]_{\mathrm{max}}$$
$$K_{\mathrm{LA}}^{\mathrm{AAB}}=Ks_5\frac{[\mathrm{LA}]_{\mathrm{max}}}{[\mathrm{AAB}]_{\mathrm{max}}}$$
$$k_{\mathrm{Y}}=\frac{k_1}{[\mathrm{EtOH}]_{\mathrm{max}}}$$
$$k_{\mathrm{LAB}}=\frac{k_2}{[\mathrm{LA}]_{\mathrm{max}}}$$

$$k_{\mathrm{AAB}} = \frac{k_3}{[\mathrm{Ac}]_{\max}^2} \qquad Y_{\mathrm{EtOH|AAB}} = Y_6\,\frac{[\mathrm{EtOH}]_{\max}}{[\mathrm{AAB}]_{\max}}$$

$$Y_{\mathrm{Glc|Y}} = Y_1\,\frac{[\mathrm{Glc}]_{\max}}{[\mathrm{Y}]_{\max}} \qquad Y_{\mathrm{LA|LAB}} = Y_7\,\frac{[\mathrm{LA}]_{\max}}{[\mathrm{LAB}]_{\max}}$$

$$Y_{\mathrm{Glc|LAB}} = Y_2\,\frac{[\mathrm{Glc}]_{\max}}{[\mathrm{LAB}]_{\max}} \qquad Y_{\mathrm{LA|AAB}} = Y_8\,\frac{[\mathrm{LA}]_{\max}}{[\mathrm{AAB}]_{\max}}$$

$$Y_{\mathrm{Fru|Y}} = Y_3\,\frac{[\mathrm{Fru}]_{\max}}{[\mathrm{Y}]_{\max}} \qquad Y_{\mathrm{Ac|LAB}} = Y_9\,\frac{[\mathrm{Ac}]_{\max}}{[\mathrm{LAB}]_{\max}}$$

$$Y^{\mathrm{Glc}}_{\mathrm{EtOH|Y}} = Y_4\,\frac{[\mathrm{EtOH}]_{\max}}{[\mathrm{Y}]_{\max}} \qquad Y^{\mathrm{EtOH}}_{\mathrm{Ac|AAB}} = Y_{10}\,\frac{[\mathrm{Ac}]_{\max}}{[\mathrm{AAB}]_{\max}}$$

$$Y^{\mathrm{Fru}}_{\mathrm{EtOH|Y}} = Y_5\,\frac{[\mathrm{EtOH}]_{\max}}{[\mathrm{Y}]_{\max}} \qquad Y^{\mathrm{LA}}_{\mathrm{Ac|AAB}} = Y_{11}\,\frac{[\mathrm{Ac}]_{\max}}{[\mathrm{AAB}]_{\max}}$$

In this way, the prior θ in equation (2.13.MM) is equivalent to sample from an unscaled prior distribution determined by the conversion factors above derived. For each of the k parameters we then have

$$\theta_{k_u} \sim \mathcal{N}(c_k\,0.5,\, c_k\,0.3),\;\; \theta_{k_u} > 0, \tag{22}$$

where θ_{k_u} represents the unscaled prior distribution for parameter k in θ and c is the conversion factor for each k parameter.

In other words, Eq. (22) defines the original unscaled ranges of the parameters in which the scaled priors for θ in equation (2.13.MM) are equivalently sampled. A summary of the original ranges, from which the parameters are sampled, is given as unscaled prior distributions in Table S3.

Table S3: Prior distributions in the range of the original units of the parameters of the cocoa bean fermentation model for the data of Camu *et al.* [11] and the fermentation boxes 1 and 2 of Papalexandratou *et al.* [15]. All priors are constrained in the positive set of real numbers.

Parameter	Camu	P. Box 1	P. Box 2
$\mu_{\max}^{\mathrm{Y_{Glc}}}$	$\mathcal{N}(0.5, 0.3)$	$\mathcal{N}(0.5, 0.3)$	$\mathcal{N}(0.5, 0.3)$
$\mu_{\max}^{\mathrm{Y_{Fru}}}$	$\mathcal{N}(0.5, 0.3)$	$\mathcal{N}(0.5, 0.3)$	$\mathcal{N}(0.5, 0.3)$
$\mu_{\max}^{\mathrm{LAB}}$	$\mathcal{N}(0.5, 0.3)$	$\mathcal{N}(0.5, 0.3)$	$\mathcal{N}(0.5, 0.3)$
$\mu_{\max}^{\mathrm{AAB_{EtOH}}}$	$\mathcal{N}(0.5, 0.3)$	$\mathcal{N}(0.5, 0.3)$	$\mathcal{N}(0.5, 0.3)$
$\mu_{\max}^{\mathrm{AAB_{LA}}}$	$\mathcal{N}(0.5, 0.3)$	$\mathcal{N}(0.5, 0.3)$	$\mathcal{N}(0.5, 0.3)$
$K_{\mathrm{Glc}}^{\mathrm{Y}}$	$\mathcal{N}(25.9, 15.6)$	$\mathcal{N}(27.7, 16.6)$	$\mathcal{N}(21.5, 12.9)$
$K_{\mathrm{Fru}}^{\mathrm{Y}}$	$\mathcal{N}(28.9, 17.3)$	$\mathcal{N}(24.8, 14.9)$	$\mathcal{N}(33.6, 20.2)$
$K_{\mathrm{Glc}}^{\mathrm{LAB}}$	$\mathcal{N}(25.9, 15.6)$	$\mathcal{N}(27.7, 16.6)$	$\mathcal{N}(21.5, 12.9)$
$K_{\mathrm{EtOH}}^{\mathrm{AAB}}$	$\mathcal{N}(11.2, 6.8)$	$\mathcal{N}(3.0, 1.8)$	$\mathcal{N}(3.5, 2.1)$
$K_{\mathrm{LA}}^{\mathrm{AAB}}$	$\mathcal{N}(2243.7, 1346.2)$	$\mathcal{N}(286.2, 171.7)$	$\mathcal{N}(76.4, 45.8)$
k_{Y}	$\mathcal{N}(0.02, 0.01)$	$\mathcal{N}(0.08, 0.05)$	$\mathcal{N}(0.07, 0.04)$
k_{LAB}	$\mathcal{N}(0.06, 0.03)$	$\mathcal{N}(0.10, 0.06)$	$\mathcal{N}(0.12, 0.07)$
k_{AAB}	$\mathcal{N}(0.01, 0.008)$	$\mathcal{N}(0.003, 0.002)$	$\mathcal{N}(0.001, 0.0009)$
$Y_{\mathrm{Glc\|Y}}$	$\mathcal{N}(27.2, 16.3)$	$\mathcal{N}(130.6, 78.4)$	$\mathcal{N}(88.2, 52.9)$
$Y_{\mathrm{Glc\|LAB}}$	$\mathcal{N}(45.9, 27.5)$	$\mathcal{N}(74.2, 44.5)$	$\mathcal{N}(30.7, 18.4)$
$Y_{\mathrm{Fru\|Y}}$	$\mathcal{N}(30.2, 18.1)$	$\mathcal{N}(116.9, 70.2)$	$\mathcal{N}(138.2, 82.9)$
$Y_{\mathrm{EtOH\|Y}}^{\mathrm{Glc}}$	$\mathcal{N}(11.8, 7.1)$	$\mathcal{N}(14.2, 8.5)$	$\mathcal{N}(14.5, 8.7)$
$Y_{\mathrm{EtOH\|Y}}^{\mathrm{Fru}}$	$\mathcal{N}(11.8, 7.1)$	$\mathcal{N}(14.2, 8.5)$	$\mathcal{N}(14.5, 8.7)$
$Y_{\mathrm{EtOH\|AAB}}$	$\mathcal{N}(5872.7, 3523.6)$	$\mathcal{N}(354.3, 212.6)$	$\mathcal{N}(130.7, 78.4)$
$Y_{\mathrm{LA\|LAB}}$	$\mathcal{N}(7.6, 4.6)$	$\mathcal{N}(6.5, 3.9)$	$\mathcal{N}(2.9, 1.8)$
$Y_{\mathrm{LA\|AAB}}$	$\mathcal{N}(2243.7, 1346.2)$	$\mathcal{N}(286.2, 171.7)$	$\mathcal{N}(76.4, 45.8)$
$Y_{\mathrm{Ac\|LAB}}$	$\mathcal{N}(5.4, 3.3)$	$\mathcal{N}(15.9, 9.6)$	$\mathcal{N}(13.3, 7.9)$
$Y_{\mathrm{Ac\|AAB}}^{\mathrm{EtOH}}$	$\mathcal{N}(1602.9, 961.7)$	$\mathcal{N}(700.9, 420.5)$	$\mathcal{N}(345.1, 207.0)$
$Y_{\mathrm{Ac\|AAB}}^{\mathrm{LA}}$	$\mathcal{N}(1602.9, 961.7)$	$\mathcal{N}(700.9, 420.5)$	$\mathcal{N}(345.1, 207.0)$

Supplementary Material S4: Model's diagnostics

Table S4: Scaled posterior moments and quantiles of parameter estimates for the Camu *et al.* [11] dataset. Number of effective sample size and $\hat{R}$ statistic are also shown.

Parameter	Mean	SE mean	sd	2.5%	25%	50%	75%	97.5%	n-eff	$\hat{R}$
$\mu_{\mathrm{max}}^{\mathrm{Y_{Glc}}}$	0.253	0.002	0.094	0.098	0.184	0.242	0.314	0.465	3369	1.00
$\mu_{\mathrm{max}}^{\mathrm{Y_{Fru}}}$	0.358	0.002	0.106	0.173	0.282	0.352	0.429	0.584	4280	1.00
$\mu_{\mathrm{max}}^{\mathrm{LAB}}$	0.358	0.001	0.067	0.237	0.311	0.354	0.402	0.500	5427	1.00
$\mu_{\mathrm{max}}^{\mathrm{AAB_{EtOH}}}$	0.380	0.001	0.092	0.214	0.317	0.376	0.437	0.577	4194	1.00
$\mu_{\mathrm{max}}^{\mathrm{AAB_{LA}}}$	0.008	0.000	0.012	0.000	0.002	0.005	0.009	0.038	1597	1.00
$K_{\mathrm{Glc}}^{\mathrm{Y}}$	0.680	0.003	0.266	0.181	0.493	0.669	0.858	1.215	8000	1.00
$K_{\mathrm{Fru}}^{\mathrm{Y}}$	0.615	0.003	0.264	0.125	0.424	0.607	0.791	1.157	6066	1.00
$K_{\mathrm{Glc}}^{\mathrm{LAB}}$	0.731	0.003	0.238	0.294	0.560	0.718	0.887	1.220	5931	1.00
$K_{\mathrm{EtOH}}^{\mathrm{AAB}}$	0.714	0.003	0.251	0.252	0.539	0.705	0.879	1.236	6190	1.00
$K_{\mathrm{LA}}^{\mathrm{AAB}}$	0.559	0.003	0.275	0.076	0.358	0.544	0.748	1.132	8000	1.00
k_{Y}	0.748	0.002	0.114	0.544	0.669	0.741	0.820	0.989	5105	1.00
k_{LAB}	0.047	0.000	0.012	0.028	0.038	0.045	0.053	0.076	3816	1.00
k_{AAB}	0.259	0.001	0.053	0.167	0.223	0.255	0.292	0.377	5838	1.00
$Y_{\mathrm{Glc\|Y}}$	0.614	0.003	0.207	0.275	0.461	0.592	0.745	1.057	4527	1.00
$Y_{\mathrm{Glc\|LAB}}$	0.319	0.002	0.118	0.128	0.235	0.304	0.390	0.588	4277	1.00
$Y_{\mathrm{Fru\|Y}}$	0.680	0.002	0.185	0.382	0.544	0.658	0.796	1.085	5325	1.00
$Y_{\mathrm{EtOH\|Y}}^{\mathrm{Glc}}$	0.316	0.003	0.192	0.025	0.169	0.295	0.438	0.747	5586	1.00
$Y_{\mathrm{EtOH\|Y}}^{\mathrm{Fru}}$	0.252	0.002	0.142	0.024	0.145	0.238	0.342	0.569	5744	1.00
$Y_{\mathrm{EtOH\|AAB}}$	0.110	0.001	0.054	0.039	0.076	0.101	0.133	0.236	2407	1.00
$Y_{\mathrm{LA\|LAB}}$	0.700	0.002	0.102	0.517	0.628	0.695	0.763	0.919	4186	1.00
$Y_{\mathrm{LA\|AAB}}$	0.430	0.003	0.271	0.022	0.215	0.402	0.614	1.010	8000	1.00
$Y_{\mathrm{Ac\|LAB}}$	0.517	0.001	0.085	0.363	0.458	0.513	0.573	0.698	4553	1.00
$Y_{\mathrm{Ac\|AAB}}^{\mathrm{EtOH}}$	0.032	0.001	0.031	0.001	0.013	0.026	0.044	0.097	2640	1.00
$Y_{\mathrm{Ac\|AAB}}^{\mathrm{LA}}$	0.445	0.004	0.270	0.026	0.233	0.423	0.625	1.030	5728	1.00
σ	0.149	0.000	0.010	0.131	0.142	0.148	0.156	0.171	8000	1.00

Table S5: Scaled posterior moments and quantiles of parameter estimates for the Papalexandratou *et al.* [15] dataset of Box 1. Number of effective sample size and $\hat{R}$ statistic are also shown.

Parameter	Mean	SE mean	sd	2.5%	25%	50%	75%	97.5%	n-eff	$\hat{R}$
$\mu_{\max}^{\mathrm{Y_{Glc}}}$	0.063	0.000	0.025	0.024	0.046	0.060	0.077	0.122	5307	1.00
$\mu_{\max}^{\mathrm{Y_{Fru}}}$	0.083	0.000	0.030	0.038	0.062	0.079	0.100	0.154	5569	1.00
$\mu_{\max}^{\mathrm{LAB}}$	0.414	0.001	0.096	0.246	0.346	0.408	0.477	0.617	4494	1.00
$\mu_{\max}^{\mathrm{AAB_{EtOH}}}$	0.150	0.001	0.051	0.061	0.113	0.148	0.184	0.258	4873	1.00
$\mu_{\max}^{\mathrm{AAB_{LA}}}$	0.025	0.000	0.017	0.002	0.012	0.021	0.034	0.065	4384	1.00
$K_{\mathrm{Glc}}^{\mathrm{Y}}$	0.619	0.003	0.270	0.123	0.425	0.615	0.800	1.170	8000	1.00
$K_{\mathrm{Fru}}^{\mathrm{Y}}$	0.504	0.004	0.286	0.040	0.281	0.483	0.697	1.101	5535	1.00
$K_{\mathrm{Glc}}^{\mathrm{LAB}}$	0.571	0.004	0.250	0.123	0.390	0.558	0.737	1.093	4238	1.00
$K_{\mathrm{EtOH}}^{\mathrm{AAB}}$	0.633	0.003	0.271	0.125	0.440	0.628	0.823	1.170	8000	1.00
$K_{\mathrm{LA}}^{\mathrm{AAB}}$	0.545	0.003	0.267	0.074	0.350	0.531	0.721	1.104	8000	1.00
k_{Y}	0.312	0.001	0.072	0.203	0.261	0.302	0.352	0.479	5425	1.00
k_{LAB}	0.310	0.002	0.089	0.159	0.250	0.304	0.362	0.509	3086	1.00
k_{AAB}	0.061	0.000	0.027	0.022	0.042	0.056	0.076	0.130	4145	1.00
$Y_{\mathrm{Glc\|Y}}$	0.922	0.003	0.229	0.495	0.765	0.911	1.072	1.390	8000	1.00
$Y_{\mathrm{Glc\|LAB}}$	0.136	0.002	0.089	0.017	0.073	0.118	0.177	0.362	3166	1.00
$Y_{\mathrm{Fru\|Y}}$	1.044	0.002	0.198	0.689	0.904	1.034	1.172	1.456	8000	1.00
$Y_{\mathrm{EtOH\|Y}}^{\mathrm{Glc}}$	0.420	0.003	0.213	0.052	0.261	0.406	0.562	0.865	5429	1.00
$Y_{\mathrm{EtOH\|Y}}^{\mathrm{Fru}}$	0.394	0.003	0.176	0.063	0.267	0.393	0.515	0.745	4619	1.00
$Y_{\mathrm{EtOH\|AAB}}$	0.534	0.003	0.215	0.169	0.380	0.517	0.671	0.996	5536	1.00
$Y_{\mathrm{LA\|LAB}}$	0.214	0.001	0.051	0.137	0.179	0.207	0.241	0.333	3420	1.00
$Y_{\mathrm{LA\|AAB}}$	0.502	0.003	0.247	0.078	0.321	0.483	0.662	1.024	8000	1.00
$Y_{\mathrm{Ac\|LAB}}$	0.103	0.000	0.035	0.044	0.079	0.098	0.123	0.182	4363	1.00
$Y_{\mathrm{Ac\|AAB}}^{\mathrm{EtOH}}$	0.411	0.003	0.194	0.075	0.270	0.399	0.536	0.820	4561	1.00
$Y_{\mathrm{Ac\|AAB}}^{\mathrm{LA}}$	0.476	0.003	0.255	0.040	0.287	0.458	0.644	1.014	8000	1.00
σ	0.168	0.000	0.014	0.143	0.158	0.167	0.176	0.198	8000	1.00

Table S6: Scaled posterior moments and quantiles of parameter estimates for the Papalexandratou *et al.* [15] dataset of Box 2. Number of effective sample size and $\hat{R}$ statistic are also shown.

Parameter	Mean	SE mean	sd	2.5%	25%	50%	75%	97.5%	n-eff	$\hat{R}$
$\mu_{\max}^{Y_{\mathrm{Glc}}}$	0.368	0.002	0.131	0.162	0.269	0.354	0.447	0.664	3979	1.00
$\mu_{\max}^{Y_{\mathrm{Fru}}}$	0.572	0.002	0.151	0.301	0.465	0.562	0.671	0.887	4323	1.00
$\mu_{\max}^{\mathrm{LAB}}$	0.499	0.002	0.152	0.227	0.388	0.492	0.600	0.815	4436	1.00
$\mu_{\max}^{\mathrm{AAB}_{\mathrm{EtOH}}}$	0.168	0.001	0.052	0.079	0.130	0.165	0.202	0.280	4578	1.00
$\mu_{\max}^{\mathrm{AAB}_{\mathrm{LA}}}$	0.022	0.000	0.016	0.001	0.009	0.019	0.031	0.060	4019	1.00
$K_{\mathrm{Glc}}^{\mathrm{Y}}$	0.699	0.003	0.254	0.231	0.519	0.688	0.867	1.213	8000	1.00
$K_{\mathrm{Fru}}^{\mathrm{Y}}$	0.615	0.003	0.257	0.132	0.434	0.609	0.788	1.142	6094	1.00
$K_{\mathrm{Glc}}^{\mathrm{LAB}}$	0.449	0.004	0.247	0.046	0.263	0.430	0.615	0.972	4583	1.00
$K_{\mathrm{EtOH}}^{\mathrm{AAB}}$	0.575	0.004	0.281	0.073	0.369	0.567	0.767	1.143	5840	1.00
$K_{\mathrm{LA}}^{\mathrm{AAB}}$	0.537	0.003	0.271	0.064	0.334	0.528	0.715	1.101	8000	1.00
k_{Y}	0.649	0.003	0.175	0.343	0.519	0.644	0.772	0.994	3747	1.00
k_{LAB}	0.283	0.002	0.103	0.106	0.210	0.275	0.347	0.510	3237	1.00
k_{AAB}	0.128	0.001	0.053	0.053	0.091	0.119	0.155	0.255	5813	1.00
$Y_{\mathrm{Glc}\|\mathrm{Y}}$	0.678	0.003	0.199	0.350	0.532	0.660	0.804	1.109	5925	1.00
$Y_{\mathrm{Glc}\|\mathrm{LAB}}$	0.054	0.001	0.056	0.002	0.016	0.037	0.073	0.209	4101	1.00
$Y_{\mathrm{Fru}\|\mathrm{Y}}$	0.841	0.003	0.228	0.448	0.676	0.828	0.989	1.322	6484	1.00
$Y_{\mathrm{EtOH}\|\mathrm{Y}}^{\mathrm{Glc}}$	0.283	0.002	0.155	0.025	0.168	0.271	0.384	0.621	4052	1.00
$Y_{\mathrm{EtOH}\|\mathrm{Y}}^{\mathrm{Fru}}$	0.207	0.002	0.134	0.011	0.104	0.190	0.292	0.507	4731	1.00
$Y_{\mathrm{EtOH}\|\mathrm{AAB}}$	0.652	0.003	0.215	0.296	0.496	0.630	0.787	1.132	6241	1.00
$Y_{\mathrm{LA}\|\mathrm{LAB}}$	0.363	0.002	0.136	0.185	0.268	0.333	0.424	0.717	3402	1.00
$Y_{\mathrm{LA}\|\mathrm{AAB}}$	0.412	0.003	0.252	0.027	0.215	0.386	0.575	0.968	5647	1.00
$Y_{\mathrm{Ac}\|\mathrm{LAB}}$	0.109	0.001	0.071	0.011	0.060	0.095	0.142	0.286	4521	1.00
$Y_{\mathrm{Ac}\|\mathrm{AAB}}^{\mathrm{EtOH}}$	0.466	0.003	0.192	0.096	0.337	0.462	0.589	0.866	4820	1.00
$Y_{\mathrm{Ac}\|\mathrm{AAB}}^{\mathrm{LA}}$	0.558	0.003	0.257	0.083	0.377	0.551	0.730	1.080	8000	1.00
σ	0.168	0.000	0.013	0.144	0.158	0.167	0.176	0.195	8000	1.00

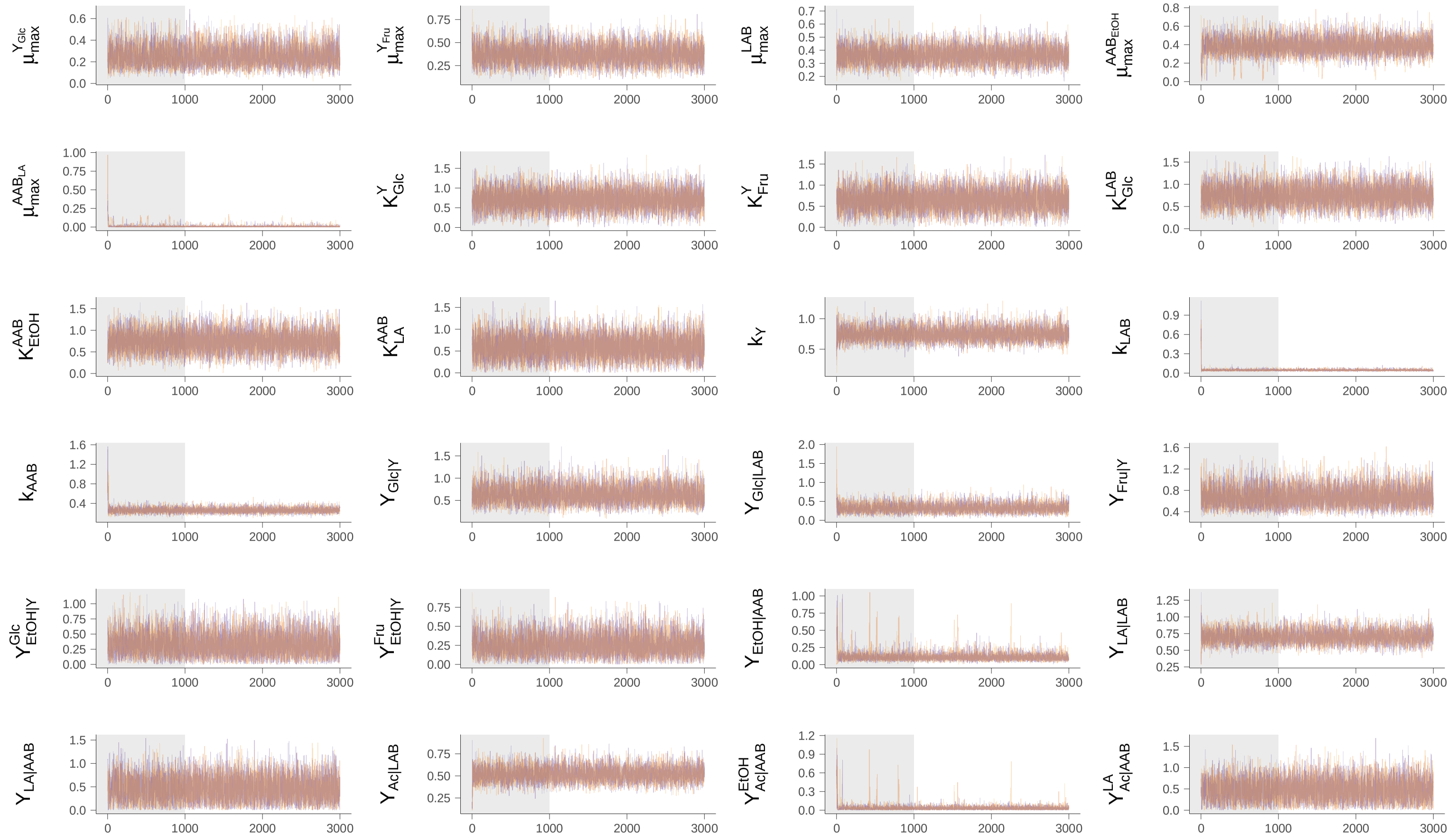

Figure S6: Scaled traceplots for the 24 parameters of the model estimated from the dataset of Camu *et al.* [11]. Different color lines, represent different MCMC. The shadowed area represents the 1000 iterations used as warm-up.

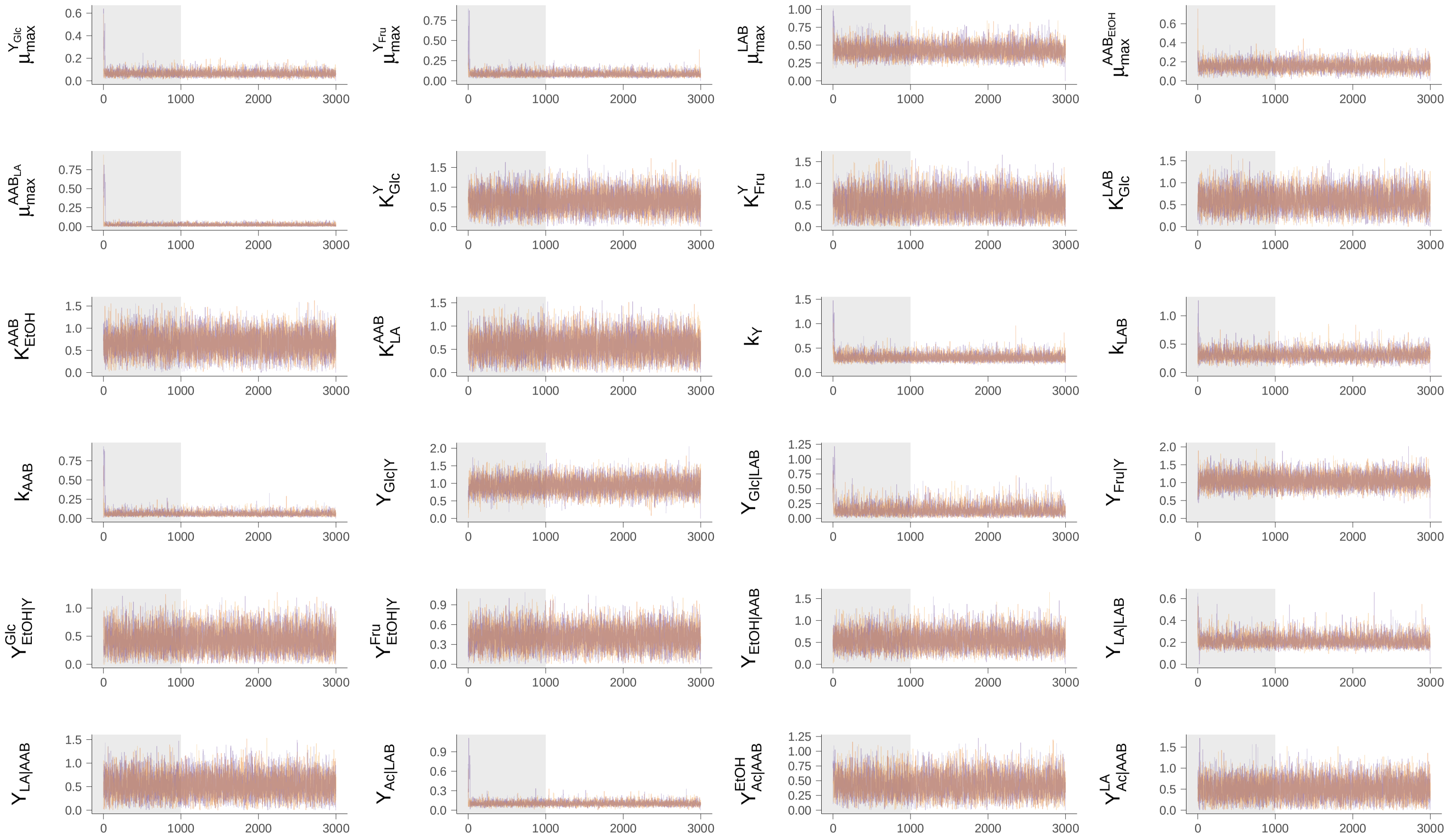

Figure S7: Scaled traceplots for the 24 parameters of the model estimated from the dataset of Box 1 of Papalexandratou *et al.* [15]. Different color lines, represent different MCMC. The shadowed area represents the 1000 iterations used as warm-up.

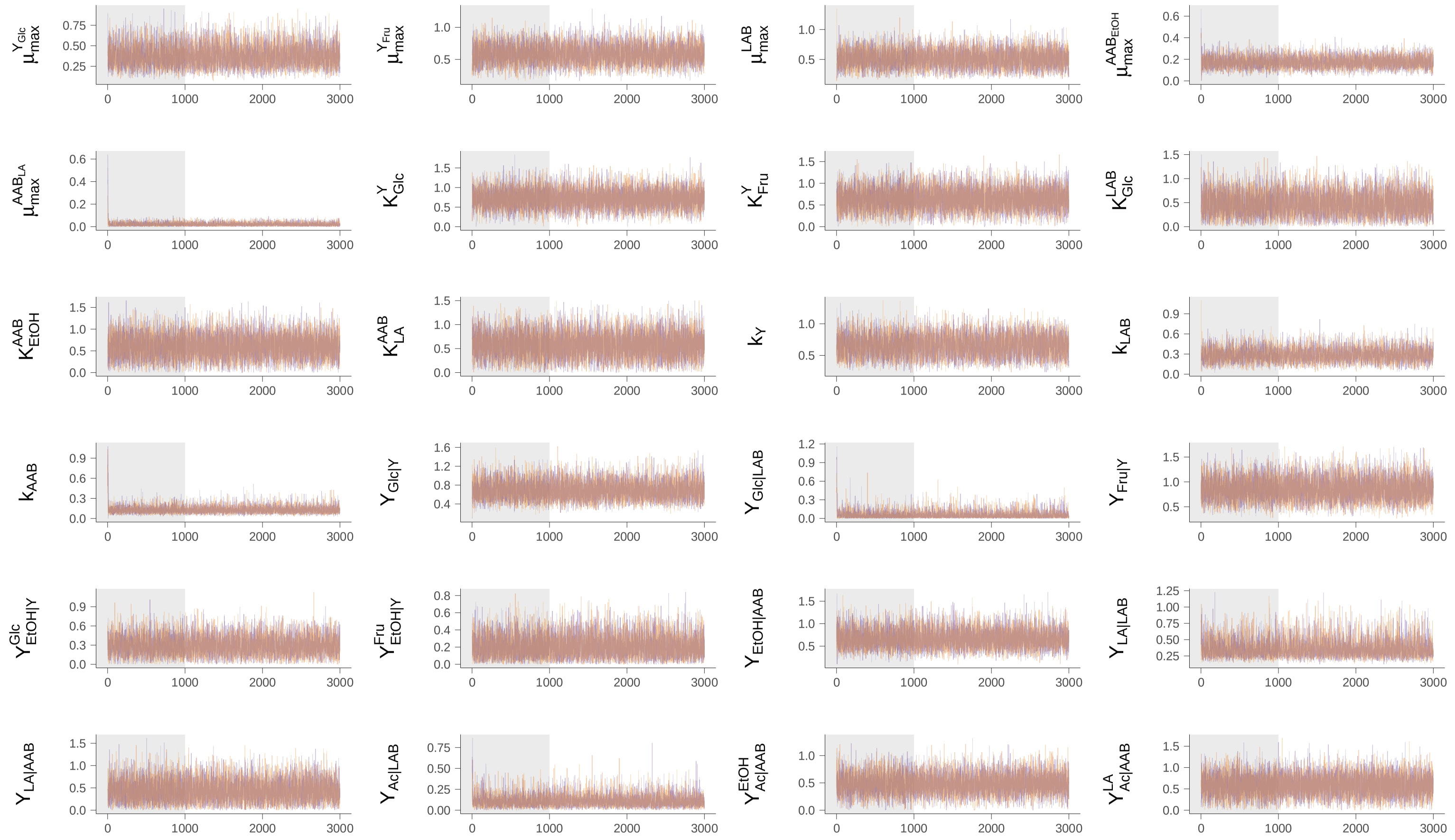

Figure S8: Scaled traceplots for the 24 parameters of the model estimated from the dataset of Box 2 of Papalexandratou *et al.* [15]. Different color lines, represent different MCMC. The shadowed area represents the 1000 iterations used as warm-up.

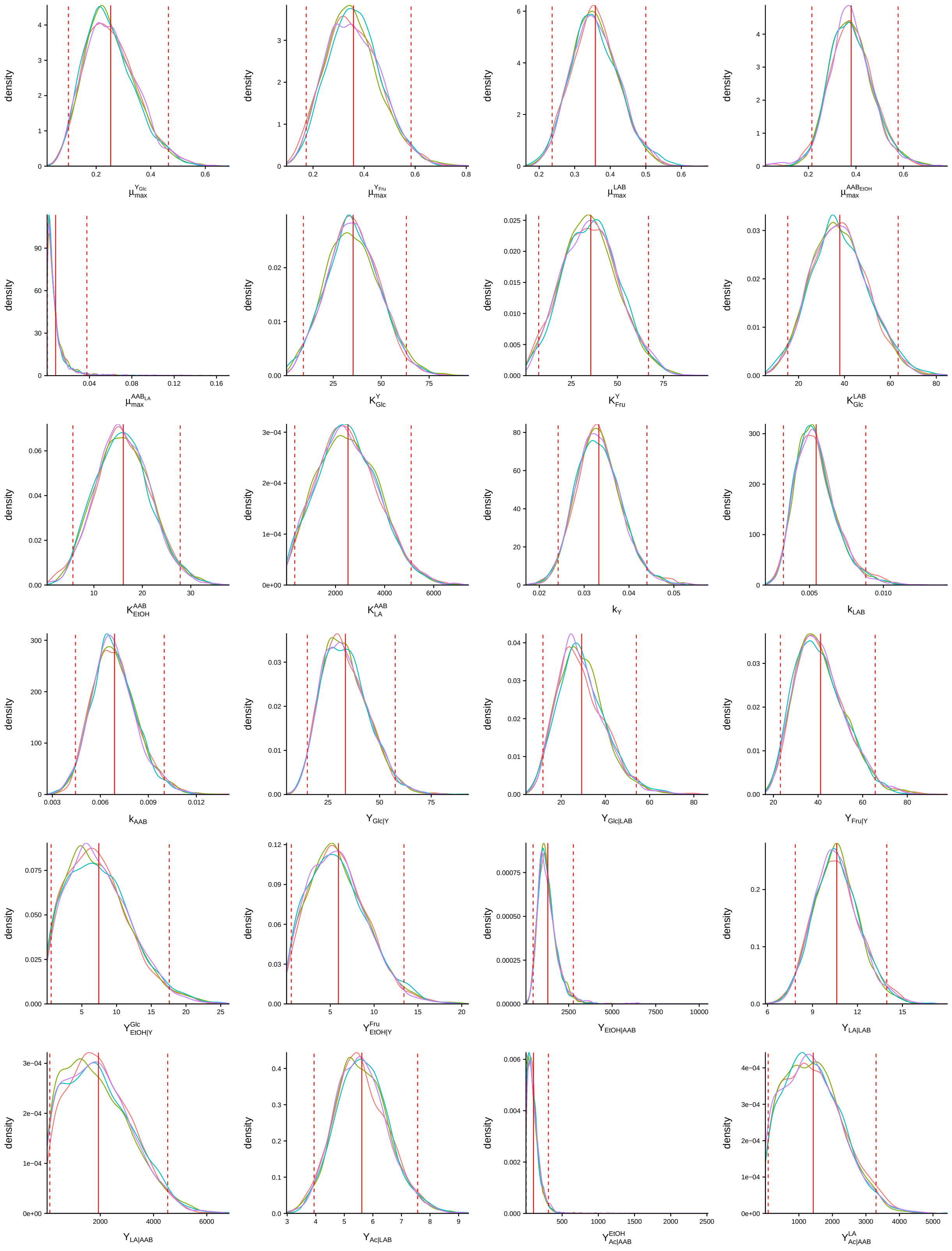

Figure S9: Unscaled posterior density distributions for the estimated parameters of the proposed models for the data reported by Camu *et al.* [11]. A different color represents a single Markov Chain. The vertical solid vertical lines represent the means of the distributions. The vertical dashed lines denote the 95% credible intervals.

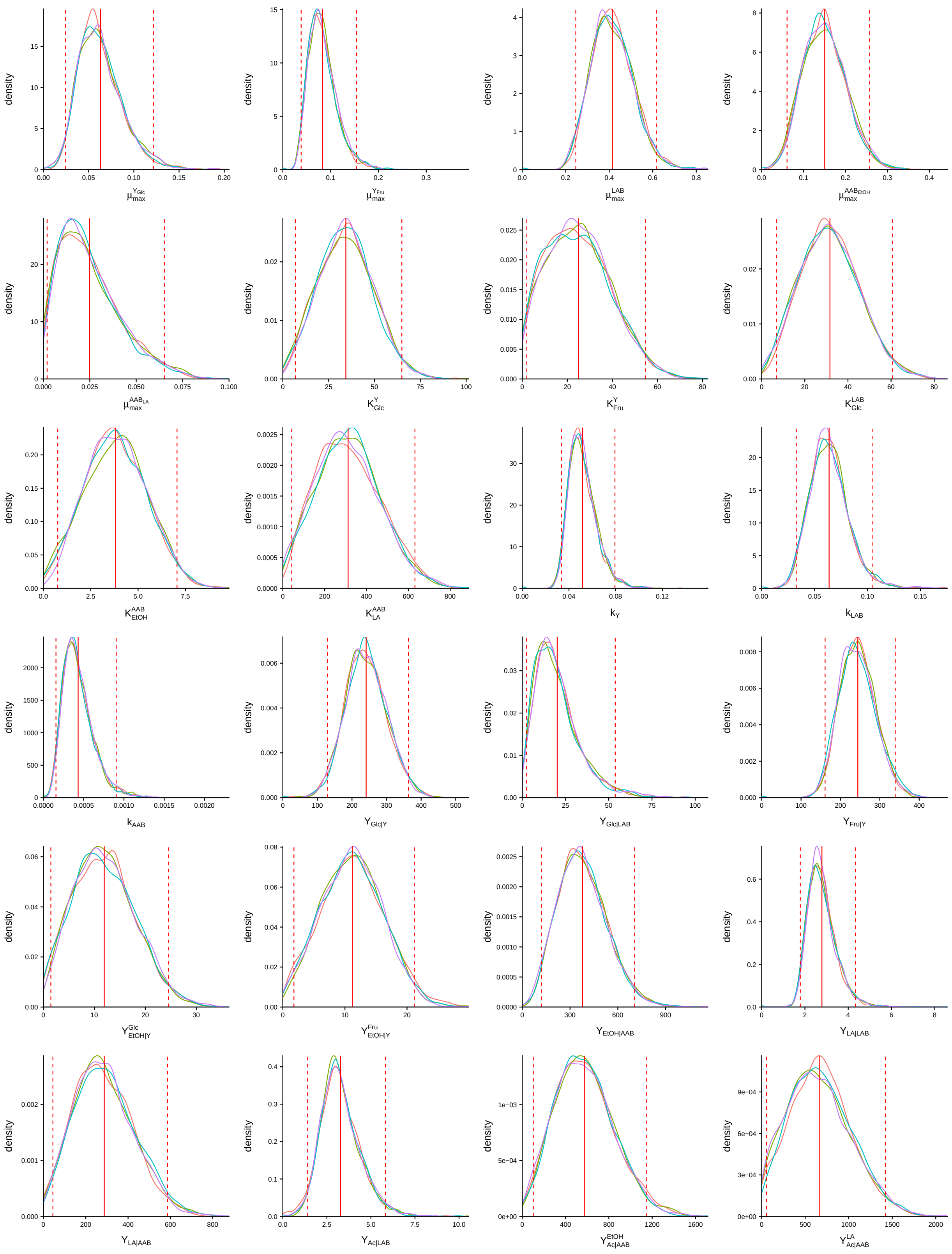

Figure S10: Unscaled posterior density distributions for the estimated parameters of the proposed models for the data reported for box 1 by Papalexandratou *et al.* [15]. A different color represents a single Markov Chain. The vertical solid vertical lines represent the means of the distributions. The vertical dashed lines denote the 95% credible intervals.

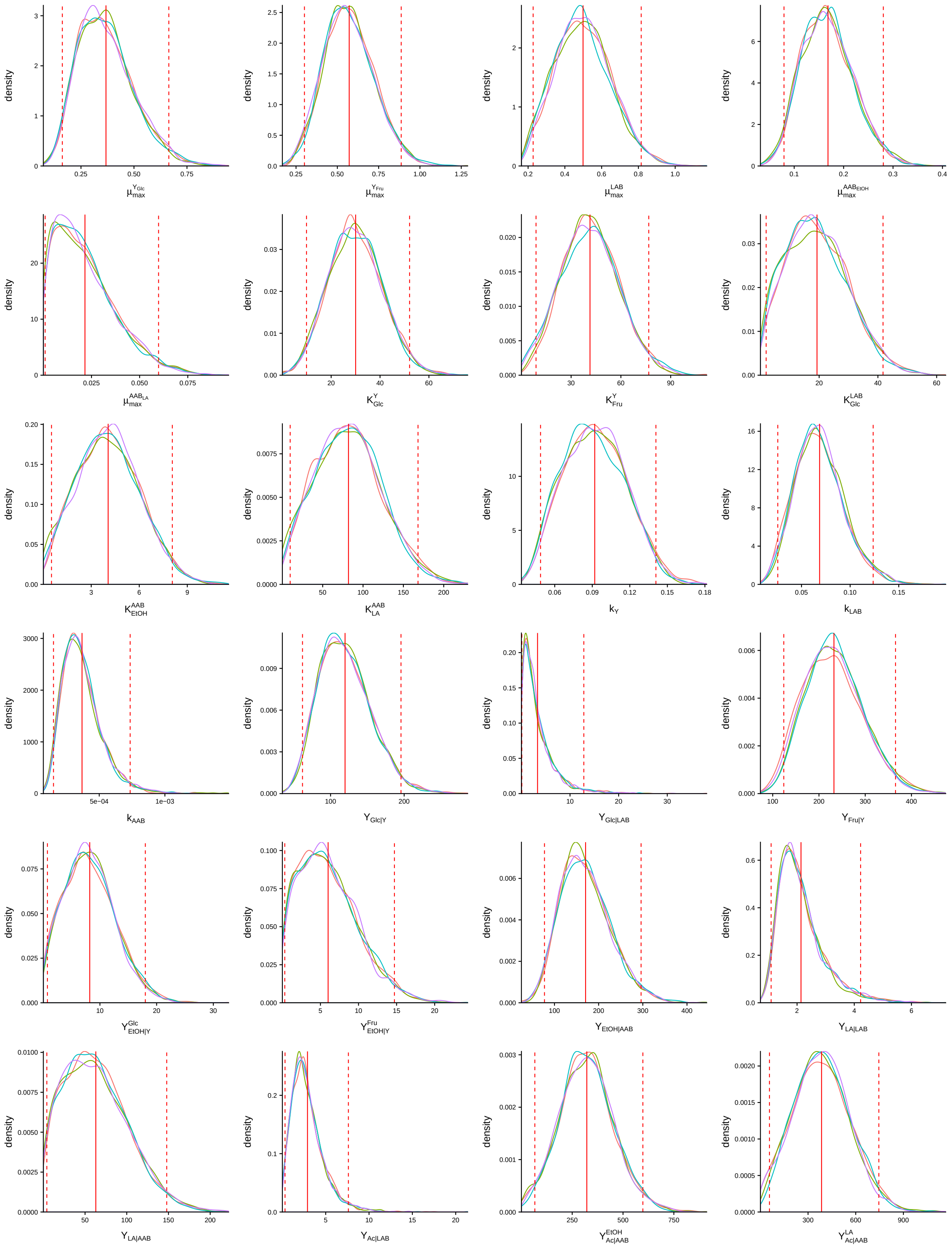

Figure S11: Unscaled posterior density distributions for the estimated parameters of the proposed models for the data reported for box 2 by Papalexandratou *et al.* [15]. A different color represents a single Markov Chain. The vertical solid vertical lines represent the means of the distributions. The vertical dashed lines denote the 95% credible intervals.

Supplementary Material S5: Asymptotic behaviour

The biologically relevant aspect of the dynamics of this fermentation model is the transient with its sequential activation of different populations. It is, however, also instructive to analyse the asymptotic behaviour of the model.

Figure S12 shows a longer time series for each of the parameter vectors derived from the three data sets.

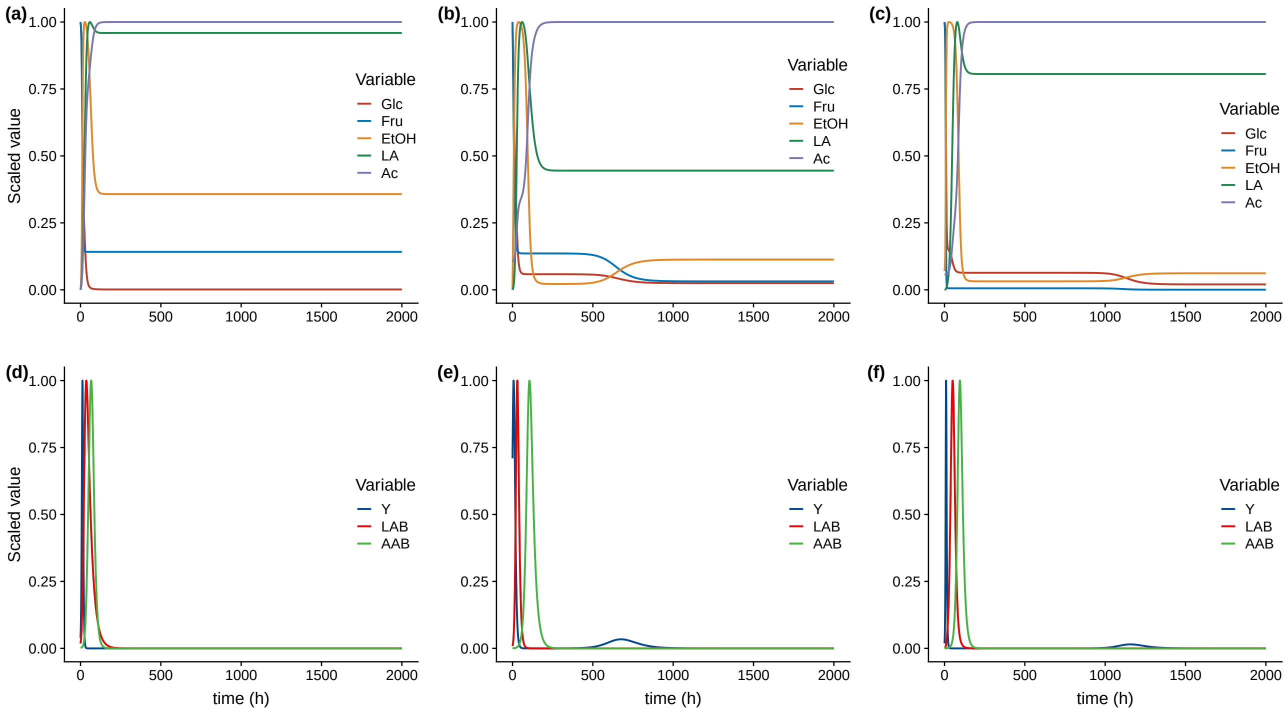

Figure S12: Long time behaviour of the model. Values scaled to one are used for visualization purposes. Metabolite kinetics for the data reported by (a) Camu *et al.* [11], (b) Box 1, Papalexandratou *et al.* [15] and (c) Box 2, Papalexandratou *et al.* [15]. Microbial community dynamics for the data reported by (d) Camu *et al.* [11], (e) Box 1, Papalexandratou *et al.* [15] and (f) Box 2, Papalexandratou *et al.* [15].

Analytically, one can see that the fixed point of the model has the following form: For some dynamical variables the fixed point value is zero, while others are undetermined or fulfil algebraic relationships, e.g.:

$$\begin{aligned}
&[\mathbf{Glc}] = [\mathrm{Glc}],\ [\mathbf{Fru}] = 0,\ [\mathbf{EtOH}] = \frac{k_{\mathrm{LAB}}[\mathrm{Glc}]}{Y_{\mathrm{Ac|LAB}}(Y^{\mathrm{Glc}}_{\mathrm{EtOH|Y}} + [\mathrm{Glc}])},\ [\mathbf{LA}] = \frac{Y_{\mathrm{Glc|Y}}[\mathrm{Glc}]}{Y^{\mathrm{EtOH}}_{\mathrm{Ac|AAB}}(Y_{\mathrm{EtOH|AAB}} + [\mathrm{Glc}])},\\
&[\mathbf{Ac}] = \frac{\sqrt{Y_{\mathrm{Fru|Y}}Y^{\mathrm{Glc}}_{\mathrm{EtOH|Y}}Y_{\mathrm{LA|LAB}}Y_{\mathrm{Ac|LAB}} + Y_{\mathrm{Fru|Y}}Y_{\mathrm{LA|LAB}}[\mathrm{Glc}]Y_{\mathrm{Ac|LAB}} + k_{\mathrm{LAB}}Y_{\mathrm{Glc|LAB}}[\mathrm{Glc}] + k_{\mathrm{LAB}}Y_{\mathrm{Fru|Y}}[\mathrm{Glc}]}}{\sqrt{Y^{\mathrm{Glc}}_{\mathrm{EtOH|Y}}Y_{\mathrm{LA|LAB}}Y_{\mathrm{Ac|LAB}}Y^{\mathrm{LA}}_{\mathrm{Ac|AAB}} + k_{\mathrm{LAB}}[\mathrm{Glc}]Y^{\mathrm{LA}}_{\mathrm{Ac|AAB}} + Y_{\mathrm{LA|LAB}}Y_{\mathrm{Ac|LAB}}[\mathrm{Glc}]Y^{\mathrm{LA}}_{\mathrm{Ac|AAB}}}},\\
&[\mathbf{Y}] = 0,\ [\mathbf{LAB}] = 0,\ [\mathbf{AAB}] = 0
\end{aligned} \tag{23}$$

as $\frac{dx_i}{dt} = 0$ is an underdetermined system.

As several combinations of variables being zero can lead to a fixed point, in total 28 such fixed points exist, all following the pattern described above. In Table S7 they are listed.

Table S7: Analytical solution for the fixed points of the model.

**[Glc]**	**[Fru]**	**[EtOH]**	**[LA]**	**[Ac]**	**[Y]**	**[LAB]**	**[AAB]**
[Glc]	[Fru]	[EtOH]	[LA]	[Ac]	0	0	0
0	[Fru]	[EtOH]	[LA]	[Ac]	0	0	0
[Glc]	[Fru]	[EtOH]	$\frac{Y_{\mathrm{Glc\vert Y}}[\mathrm{Glc}]}{Y^{\mathrm{EtOH}}_{\mathrm{Ac\vert AAB}}(Y_{\mathrm{EtOH\vert AAB}}+[\mathrm{Glc}])}$	[Ac]	0	0	0
[Glc]	[Fru]	[EtOH]	[LA]	$-\frac{\sqrt{Y_{\mathrm{Fru\vert Y}}Y_{\mathrm{LA\vert LAB}}+Y_{\mathrm{Glc\vert LAB}}[\mathrm{EtOH}]+Y_{\mathrm{Fru\vert Y}}[\mathrm{EtOH}]}}{\sqrt{Y_{\mathrm{LA\vert LAB}}Y^{\mathrm{LA}}_{\mathrm{Ac\vert AAB}}+[\mathrm{EtOH}]Y^{\mathrm{LA}}_{\mathrm{Ac\vert AAB}}}}$	0	0	0
[Glc]	[Fru]	[EtOH]	[LA]	$\frac{\sqrt{Y_{\mathrm{Fru\vert Y}}Y_{\mathrm{LA\vert LAB}}+Y_{\mathrm{Glc\vert LAB}}[\mathrm{EtOH}]+Y_{\mathrm{Fru\vert Y}}[\mathrm{EtOH}]}}{\sqrt{Y_{\mathrm{LA\vert LAB}}Y^{\mathrm{LA}}_{\mathrm{Ac\vert AAB}}+[\mathrm{EtOH}]Y^{\mathrm{LA}}_{\mathrm{Ac\vert AAB}}}}$	0	0	0
0	0	0	0	0	[Y]	[LAB]	[AAB]
0	[Fru]	0	0	0	0	[LAB]	[AAB]
0	0	0	[LA]	[Ac]	[Y]	0	0
[Glc]	[Fru]	0	0	0	0	0	[AAB]
0	[Fru]	[EtOH]	[LA]	$-\frac{\sqrt{Y_{\mathrm{Fru\vert Y}}Y_{\mathrm{LA\vert LAB}}+Y_{\mathrm{Glc\vert LAB}}[\mathrm{EtOH}]+Y_{\mathrm{Fru\vert Y}}[\mathrm{EtOH}]}}{\sqrt{Y_{\mathrm{LA\vert LAB}}Y^{\mathrm{LA}}_{\mathrm{Ac\vert AAB}}+[\mathrm{EtOH}]Y^{\mathrm{LA}}_{\mathrm{Ac\vert AAB}}}}$	0	0	0
0	[Fru]	[EtOH]	[LA]	$\frac{\sqrt{Y_{\mathrm{Fru\vert Y}}Y_{\mathrm{LA\vert LAB}}+Y_{\mathrm{Glc\vert LAB}}[\mathrm{EtOH}]+Y_{\mathrm{Fru\vert Y}}[\mathrm{EtOH}]}}{\sqrt{Y_{\mathrm{LA\vert LAB}}Y^{\mathrm{LA}}_{\mathrm{Ac\vert AAB}}+[\mathrm{EtOH}]Y^{\mathrm{LA}}_{\mathrm{Ac\vert AAB}}}}$	0	0	0
[Glc]	0	$\frac{k_{\mathrm{LAB}}[\mathrm{Glc}]}{Y_{\mathrm{Ac\vert LAB}}(Y^{\mathrm{Glc}}_{\mathrm{EtOH\vert Y}}+[\mathrm{Glc}])}$	[LA]	[Ac]	0	0	0
[Glc]	[Fru]	[EtOH]	$\frac{Y_{\mathrm{Glc\vert Y}}[\mathrm{Glc}]}{Y^{\mathrm{EtOH}}_{\mathrm{Ac\vert AAB}}(Y_{\mathrm{EtOH\vert AAB}}+[\mathrm{Glc}])}$	$-\frac{\sqrt{Y_{\mathrm{Fru\vert Y}}Y_{\mathrm{LA\vert LAB}}+Y_{\mathrm{Glc\vert LAB}}[\mathrm{EtOH}]+Y_{\mathrm{Fru\vert Y}}[\mathrm{EtOH}]}}{\sqrt{Y_{\mathrm{LA\vert LAB}}Y^{\mathrm{LA}}_{\mathrm{Ac\vert AAB}}+[\mathrm{EtOH}]Y^{\mathrm{LA}}_{\mathrm{Ac\vert AAB}}}}$	0	0	0
[Glc]	[Fru]	[EtOH]	$\frac{Y_{\mathrm{Glc\vert Y}}[\mathrm{Glc}]}{Y^{\mathrm{EtOH}}_{\mathrm{Ac\vert AAB}}(Y_{\mathrm{EtOH\vert AAB}}+[\mathrm{Glc}])}$	$\frac{\sqrt{Y_{\mathrm{Fru\vert Y}}Y_{\mathrm{LA\vert LAB}}+Y_{\mathrm{Glc\vert LAB}}[\mathrm{EtOH}]+Y_{\mathrm{Fru\vert Y}}[\mathrm{EtOH}]}}{\sqrt{Y_{\mathrm{LA\vert LAB}}Y^{\mathrm{LA}}_{\mathrm{Ac\vert AAB}}+[\mathrm{EtOH}]Y^{\mathrm{LA}}_{\mathrm{Ac\vert AAB}}}}$	0	0	0
0	0	0	0	0	[Y]	$-\frac{\mu^{\mathrm{Y\,Glc}}_{\max}k_{\mathrm{LAB}}Y_{\mathrm{EtOH\vert AAB}}[\mathrm{Y}]}{Y_{\mathrm{Glc\vert Y}}Y^{\mathrm{Glc}}_{\mathrm{EtOH\vert Y}}c_2}$	[AAB]
0	[Fru]	0	0	0	0	0	[AAB]
0	0	0	0	0	[Y]	0	[AAB]
0	[Fru]	0	0	0	0	0	[AAB]
0	0	0	[LA]	$-\frac{\sqrt{Y_{\mathrm{Fru\vert Y}}}}{\sqrt{Y^{\mathrm{LA}}_{\mathrm{Ac\vert AAB}}}}$	[Y]	0	0
0	0	0	[LA]	$\frac{\sqrt{Y_{\mathrm{Fru\vert Y}}}}{\sqrt{Y^{\mathrm{LA}}_{\mathrm{Ac\vert AAB}}}}$	[Y]	0	0
[Glc]	0	$\frac{k_{\mathrm{LAB}}[\mathrm{Glc}]}{Y_{\mathrm{Ac\vert LAB}}(Y^{\mathrm{Glc}}_{\mathrm{EtOH\vert Y}}+[\mathrm{Glc}])}$	[LA]	$-\frac{\sqrt{Y_{\mathrm{Fru\vert Y}}Y^{\mathrm{Glc}}_{\mathrm{EtOH\vert Y}}Y_{\mathrm{LA\vert LAB}}Y_{\mathrm{Ac\vert LAB}}+Y_{\mathrm{Fru\vert Y}}Y_{\mathrm{LA\vert LAB}}[\mathrm{Glc}]Y_{\mathrm{Ac\vert LAB}}+k_{\mathrm{LAB}}Y_{\mathrm{Glc\vert LAB}}[\mathrm{Glc}]+k_{\mathrm{LAB}}Y_{\mathrm{Fru\vert Y}}[\mathrm{Glc}]}}{\sqrt{Y^{\mathrm{Glc}}_{\mathrm{EtOH\vert Y}}Y_{\mathrm{LA\vert LAB}}Y_{\mathrm{Ac\vert LAB}}Y^{\mathrm{LA}}_{\mathrm{Ac\vert AAB}}+k_{\mathrm{LAB}}[\mathrm{Glc}]Y^{\mathrm{LA}}_{\mathrm{Ac\vert AAB}}+Y_{\mathrm{LA\vert LAB}}Y_{\mathrm{Ac\vert LAB}}[\mathrm{Glc}]Y^{\mathrm{LA}}_{\mathrm{Ac\vert AAB}}}}$	0	0	0
[Glc]	0	$\frac{k_{\mathrm{LAB}}[\mathrm{Glc}]}{Y_{\mathrm{Ac\vert LAB}}(Y^{\mathrm{Glc}}_{\mathrm{EtOH\vert Y}}+[\mathrm{Glc}])}$	[LA]	$\frac{\sqrt{Y_{\mathrm{Fru\vert Y}}Y^{\mathrm{Glc}}_{\mathrm{EtOH\vert Y}}Y_{\mathrm{LA\vert LAB}}Y_{\mathrm{Ac\vert LAB}}+Y_{\mathrm{Fru\vert Y}}Y_{\mathrm{LA\vert LAB}}[\mathrm{Glc}]Y_{\mathrm{Ac\vert LAB}}+k_{\mathrm{LAB}}Y_{\mathrm{Glc\vert LAB}}[\mathrm{Glc}]+k_{\mathrm{LAB}}Y_{\mathrm{Fru\vert Y}}[\mathrm{Glc}]}}{\sqrt{Y^{\mathrm{Glc}}_{\mathrm{EtOH\vert Y}}Y_{\mathrm{LA\vert LAB}}Y_{\mathrm{Ac\vert LAB}}Y^{\mathrm{LA}}_{\mathrm{Ac\vert AAB}}+k_{\mathrm{LAB}}[\mathrm{Glc}]Y^{\mathrm{LA}}_{\mathrm{Ac\vert AAB}}+Y_{\mathrm{LA\vert LAB}}Y_{\mathrm{Ac\vert LAB}}[\mathrm{Glc}]Y^{\mathrm{LA}}_{\mathrm{Ac\vert AAB}}}}$	0	0	0
[Glc]	0	$\frac{k_{\mathrm{LAB}}[\mathrm{Glc}]}{Y_{\mathrm{Ac\vert LAB}}(Y^{\mathrm{Glc}}_{\mathrm{EtOH\vert Y}}+[\mathrm{Glc}])}$	$\frac{Y_{\mathrm{Glc\vert Y}}[\mathrm{Glc}]}{Y^{\mathrm{EtOH}}_{\mathrm{Ac\vert AAB}}(Y_{\mathrm{EtOH\vert AAB}}+[\mathrm{Glc}])}$	[Ac]	0	0	0
0	0	0	0	0	0	0	[AAB]
0	0	0	[LA]	$-\frac{\sqrt{Y_{\mathrm{Fru\vert Y}}}}{\sqrt{Y^{\mathrm{LA}}_{\mathrm{Ac\vert AAB}}}}$	0	0	0
0	0	0	[LA]	$\frac{\sqrt{Y_{\mathrm{Fru\vert Y}}}}{\sqrt{Y^{\mathrm{LA}}_{\mathrm{Ac\vert AAB}}}}$	0	0	0
[Glc]	0	$\frac{k_{\mathrm{LAB}}[\mathrm{Glc}]}{Y_{\mathrm{Ac\vert LAB}}(Y^{\mathrm{Glc}}_{\mathrm{EtOH\vert Y}}+[\mathrm{Glc}])}$	$\frac{Y_{\mathrm{Glc\vert Y}}[\mathrm{Glc}]}{Y^{\mathrm{EtOH}}_{\mathrm{Ac\vert AAB}}(Y_{\mathrm{EtOH\vert AAB}}+[\mathrm{Glc}])}$	$-\frac{\sqrt{Y_{\mathrm{Fru\vert Y}}Y^{\mathrm{Glc}}_{\mathrm{EtOH\vert Y}}Y_{\mathrm{LA\vert LAB}}Y_{\mathrm{Ac\vert LAB}}+Y_{\mathrm{Fru\vert Y}}Y_{\mathrm{LA\vert LAB}}[\mathrm{Glc}]Y_{\mathrm{Ac\vert LAB}}+k_{\mathrm{LAB}}Y_{\mathrm{Glc\vert LAB}}[\mathrm{Glc}]+k_{\mathrm{LAB}}Y_{\mathrm{Fru\vert Y}}[\mathrm{Glc}]}}{\sqrt{Y^{\mathrm{Glc}}_{\mathrm{EtOH\vert Y}}Y_{\mathrm{LA\vert LAB}}Y_{\mathrm{Ac\vert LAB}}Y^{\mathrm{LA}}_{\mathrm{Ac\vert AAB}}+k_{\mathrm{LAB}}[\mathrm{Glc}]Y^{\mathrm{LA}}_{\mathrm{Ac\vert AAB}}+Y_{\mathrm{LA\vert LAB}}Y_{\mathrm{Ac\vert LAB}}[\mathrm{Glc}]Y^{\mathrm{LA}}_{\mathrm{Ac\vert AAB}}}}$	0	0	0
[Glc]	0	$\frac{k_{\mathrm{LAB}}[\mathrm{Glc}]}{Y_{\mathrm{Ac\vert LAB}}(Y^{\mathrm{Glc}}_{\mathrm{EtOH\vert Y}}+[\mathrm{Glc}])}$	$\frac{Y_{\mathrm{Glc\vert Y}}[\mathrm{Glc}]}{Y^{\mathrm{EtOH}}_{\mathrm{Ac\vert AAB}}(Y_{\mathrm{EtOH\vert AAB}}+[\mathrm{Glc}])}$	$\frac{\sqrt{Y_{\mathrm{Fru\vert Y}}Y^{\mathrm{Glc}}_{\mathrm{EtOH\vert Y}}Y_{\mathrm{LA\vert LAB}}Y_{\mathrm{Ac\vert LAB}}+Y_{\mathrm{Fru\vert Y}}Y_{\mathrm{LA\vert LAB}}[\mathrm{Glc}]Y_{\mathrm{Ac\vert LAB}}+k_{\mathrm{LAB}}Y_{\mathrm{Glc\vert LAB}}[\mathrm{Glc}]+k_{\mathrm{LAB}}Y_{\mathrm{Fru\vert Y}}[\mathrm{Glc}]}}{\sqrt{Y^{\mathrm{Glc}}_{\mathrm{EtOH\vert Y}}Y_{\mathrm{LA\vert LAB}}Y_{\mathrm{Ac\vert LAB}}Y^{\mathrm{LA}}_{\mathrm{Ac\vert AAB}}+k_{\mathrm{LAB}}[\mathrm{Glc}]Y^{\mathrm{LA}}_{\mathrm{Ac\vert AAB}}+Y_{\mathrm{LA\vert LAB}}Y_{\mathrm{Ac\vert LAB}}[\mathrm{Glc}]Y^{\mathrm{LA}}_{\mathrm{Ac\vert AAB}}}}$	0	0	0

For those cases, where positive values of all dynamical variables are compatible with the 'template' shown in Table S7, computation of the eigenvalues of the Jacobian matrix in these fixed points yields zeroes and negative values, consistent with an underdetermined system with stable fixed points.

Supplementary Material S6: Measurement errors

As mentioned in section 2.4.1 of the main manuscript, a standard deviation σ was necessary for fitting the model on each data set. Thus, the standard deviation in all three datasets, showed lower values at decreased values of the negative log-likelihood conditioned on the posterior distribution (see Figure S13). At first glance, this result might contradict the general tendency to favour the negative log-likelihood by adding variance to the posterior probabilities of the parameter estimates. However, that kind of behaviour would have been present if instead of estimating a total standard deviation for the whole model, separate standard deviations for each state variable were computed.

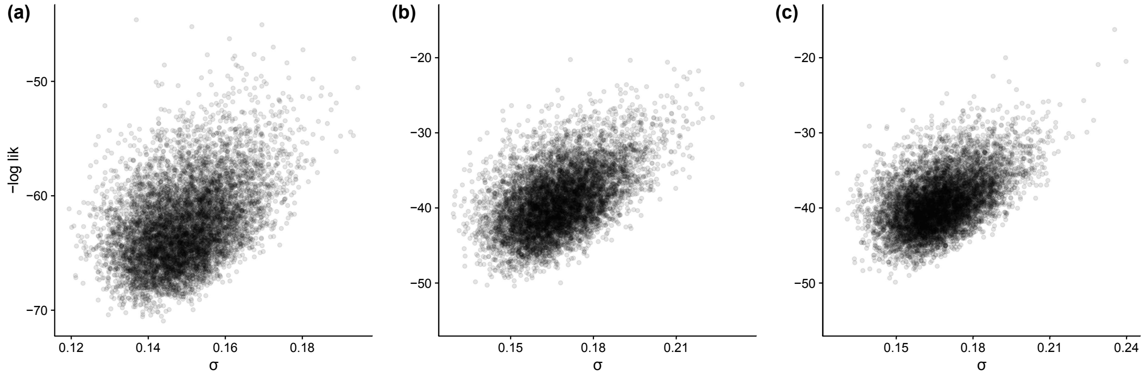

Figure S13: Scatter plots of the negative log-likelihood conditioned on the posterior distribution vs. scaled estimated standard deviation σ for each of the data sets. (a) Data reported by Camu *et al.* [11], (b) Data reported for Box 1 Papalexandratou *et al.* [15] and (c) Data reported for Box 2 Papalexandratou *et al.* [15].

To show that effect, consider the following total posterior probability:

$$P(\theta \mid \mathcal{D}) \propto \prod_{i=1}^{T} \prod_{j=1}^{N} \mathcal{N}\left(f\left(x_{i,j}, \theta\right), \sigma_i\right) P(\theta). \tag{24}$$

Eq. (24) differs from equation (2.12.MM) by σ_i, which is representing a standard deviation for each i state variable of the model. Applying Eq. (24) into our proposed model, would mean to estimate 8 standard deviations that in practice are likely to inflate the log-likelihood. As an example, by fitting a model according to Eq. (24) with the data reported by Camu *et al.* [11] the log-likelihood is increased from a mean of 62.48 obtained by the model reported in the main manuscript, to a mean of 72.91 (Figure S14).

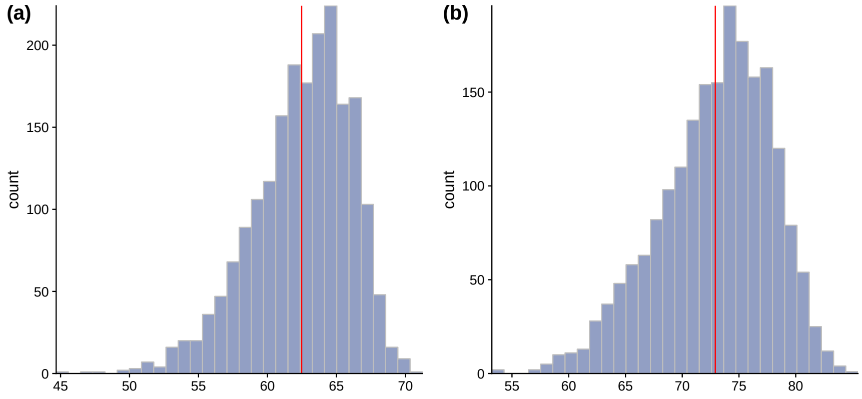

Figure S14: Log-likelihood conditioned on the posterior distribution with data from Camu *et al.* [11]. Solid vertical red line represents the mean of the distribution. (a) Model with one σ (b) Model with one σ per state variable

This increment of the log-likelihood is likely related to an inflation of certain parameters, namely $\mu_{\max}^{\mathrm{AAB}_{\mathrm{LA}}}$, $Y_{\mathrm{EtOH|AAB}}$ and $Y_{\mathrm{Ac|AAB}}^{\mathrm{LA}}$ (Figure S15).

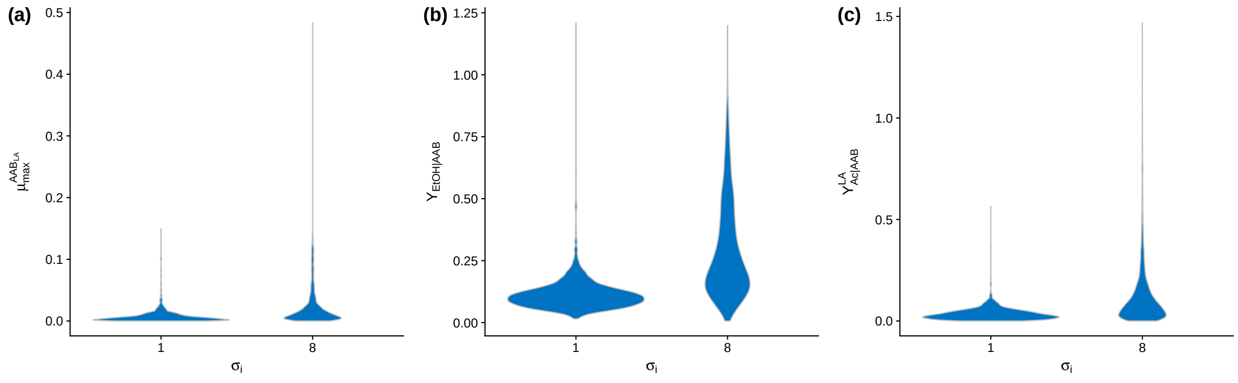

Figure S15: Posterior distributions for scaled parameters (a) $\mu_{\max}^{\mathrm{AAB}_{\mathrm{LA}}}$, (b) $Y_{\mathrm{EtOH|AAB}}$ and (c) $Y_{\mathrm{Ac|AAB}}^{\mathrm{LA}}$ with data from Camu *et al.* [11]. The x axis is representing whether the model was fitted with either one or eight σ

.

Supplementary Material S7: Parameters' correlation

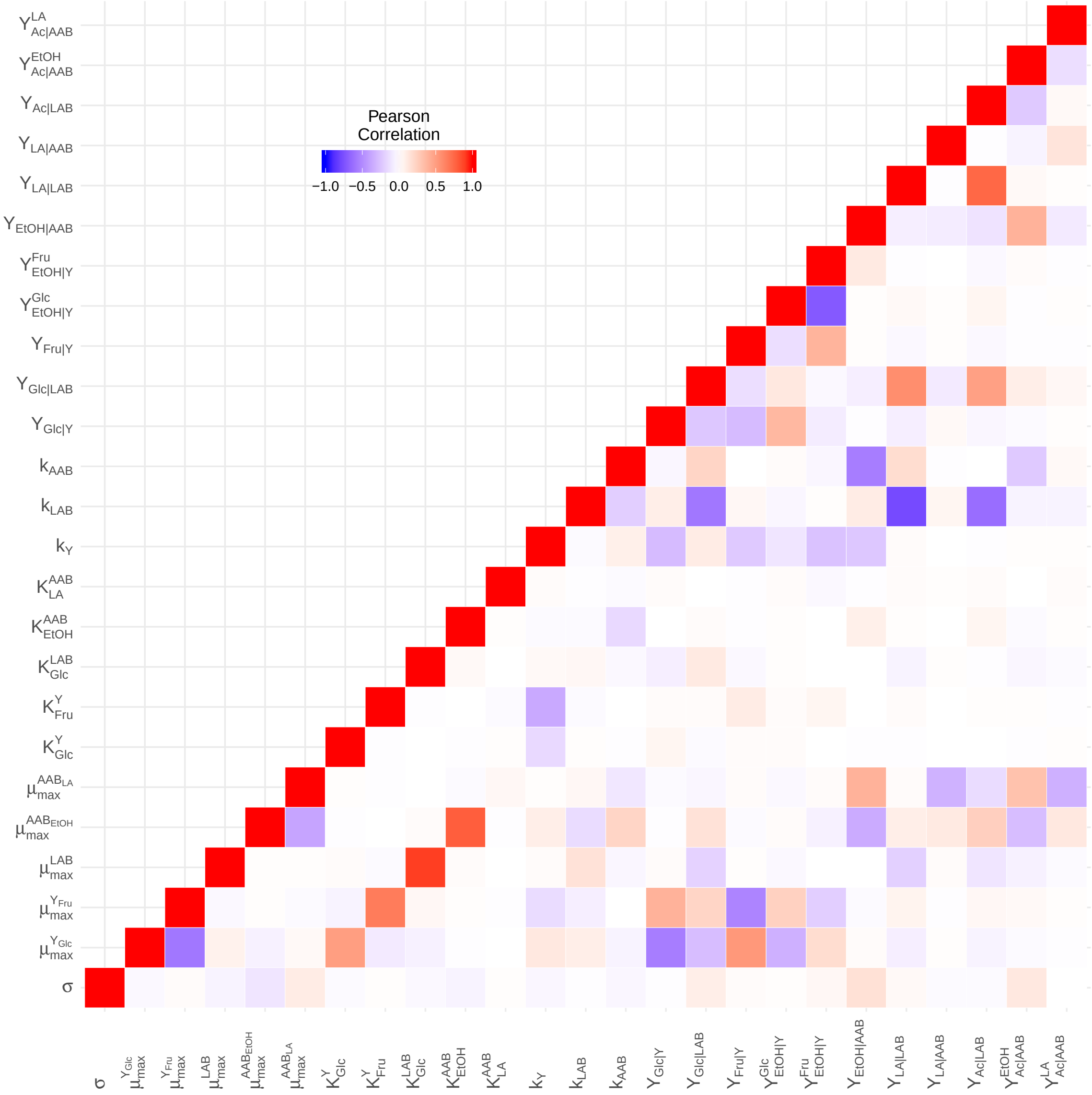

Figure S16: Heatmap based on Pearson's correlation derived from the data reported by Camu *et al.* [11].

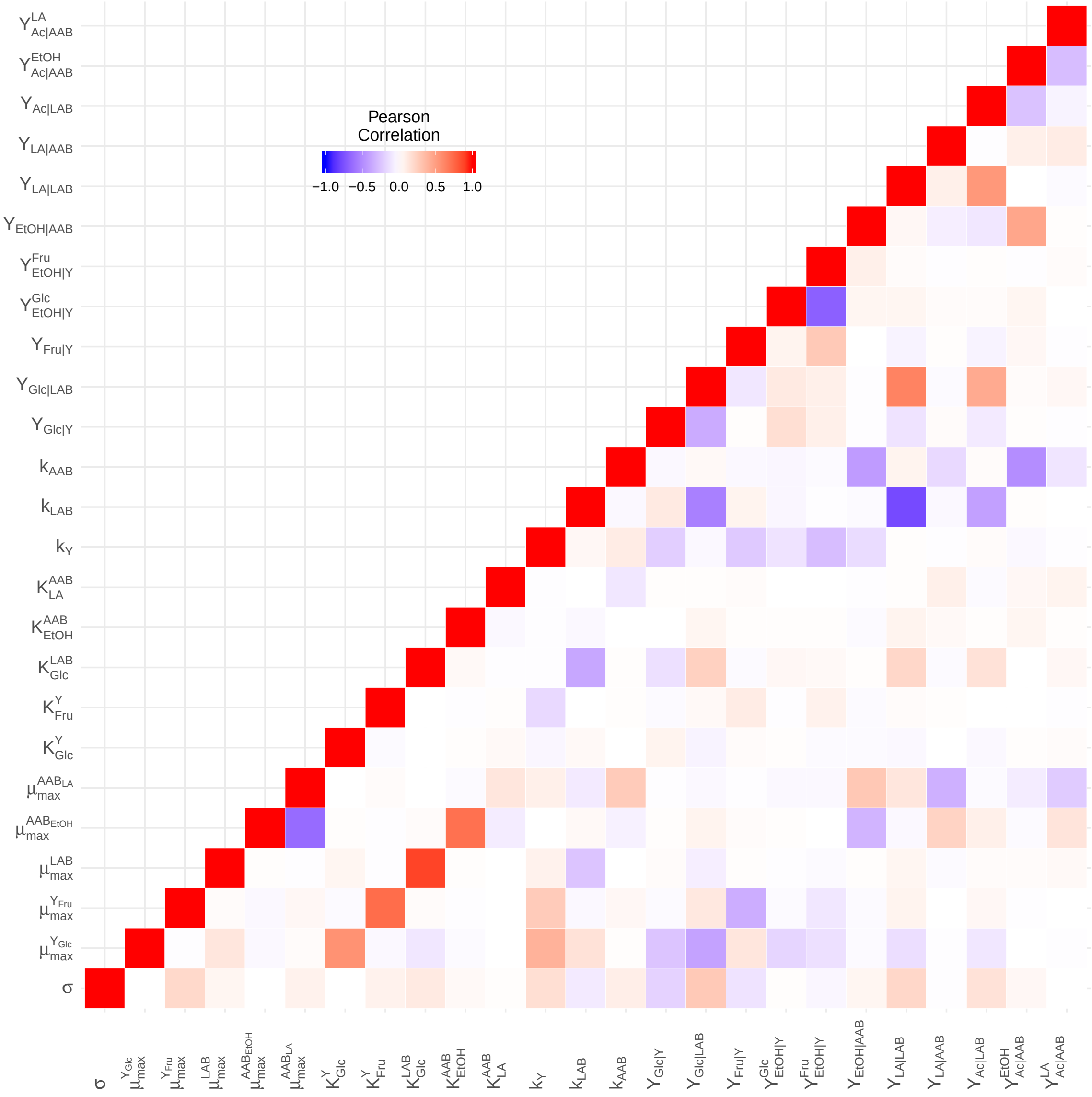

Figure S17: Heatmap based on Pearson's correlation derived from the data reported by Papalexandratou *et al.* [15] for Box 1.

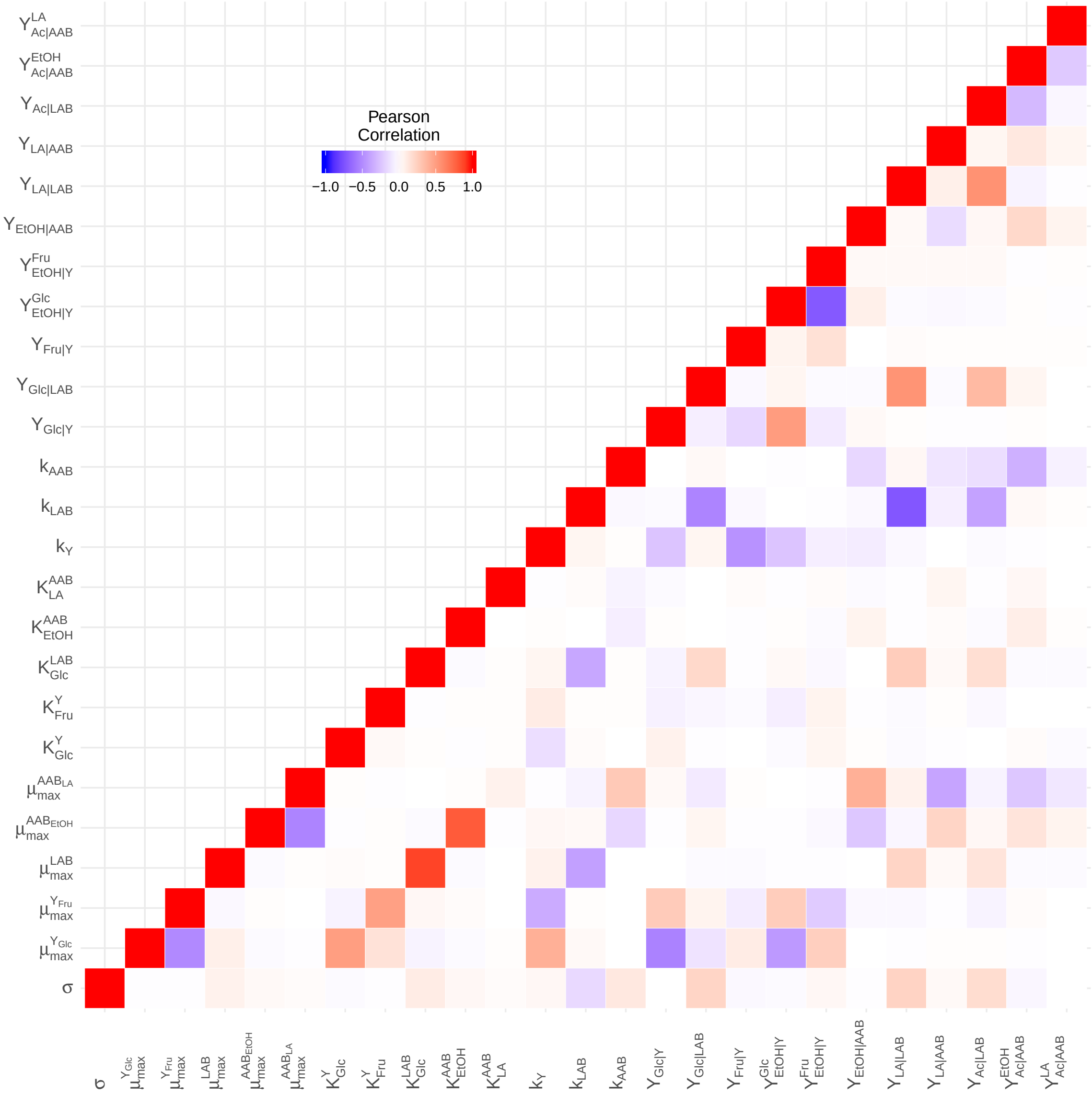

Figure S18: Heatmap based on Pearson's correlation derived from the data reported by Papalexandratou *et al.* [15] for Box 2.

References

1. Brenner, D. J., Krieg, N. R., Staley, J. T. & Garrity, G. M. (eds) 2009 *Bergey's Manual of Systematic Bacteriology: Volume 2: The proteobacteria. Part C.* Springer New York.

2. Vos, P., Garrity, G., Jones, D., Krieg, N., Ludwig, W., Rainey, F., Schleifer, K. & Whitman, W. (eds) 2009 *Bergey's Manual of Systematic Bacteriology: Volume 3: The Firmicutes.* Springer New York.

3. Neidhardt, F. C. & Umbarger, H. E. 1996 Chemical composition of *Escherichia coli.* In *Escherichia coli and Samonella typhimurium: Cellular and Molecular Biology* (eds F. C. Neidhardt, R. Curtis III, J. L. Ingraham, E. C. C. Lin, K. B. Low, B. Magasanik, R. W. S., M. Riley, D. Schneider & H. E. Umbarger), chap. 3, p. 2. ASM press, Washington, DC., 2nd edn.

4. Phillips, R., Kondev, J., Theriot, J. & Garcia, H. 2012 *Physical biology of the cell.* Taylor & Francis Group, New York, 2nd edn.

5. Monod, J. 1949 The growth of bacterial cultures. *Annual Review of Microbiology*, **3**(1), 371–394. (doi:10.1146/annurev.mi.03.100149.002103)

6. Contois, D. E. 1959 Kinetics of bacterial growth: Relationship between population density and specific growth rate of continuous cultures. *Microbiology*, **21**(1), 40–50. (doi:10.1099/00221287-21-1-40)

7. Adler, P., Frey, L. J., Berger, A., Bolten, C. J., Hansen, C. E. & Wittmann, C. 2014 The Key to Acetate: Metabolic Fluxes of Acetic Acid Bacteria under Cocoa Pulp Fermentation-Simulating Conditions. *Applied and Environmental Microbiology*, **80**(15), 4702–4716. (doi:10.1128/AEM.01048-14)

8. Moens, F., Lefeber, T. & De Vuyst, L. 2014 Oxidation of Metabolites Highlights the Microbial Interactions and Role of *Acetobacter pasteurianus* during Cocoa Bean Fermentation. *Applied and Environmental Microbiology*, **80**(6), 1848–1857. (doi:10.1128/AEM.03344-13)

9. Watson, H. E. 1908 A note on the variation of the rate of disinfection with change in the concentration of the disinfectant. *The Journal of Hygiene*, **8**(4), 536–542.

10. De Vuyst, L. & Weckx, S. 2016 The Functional Role of Lactic Acid Bacteria in Cocoa Bean Fermentation. In *Biotechnology of lactic acid bacteria* (eds F. Mozzi, R. R. Raya & G. M. Vignolo), chap. 16, pp. 248–278. Wiley Blackwell, 2nd edn.

11. Camu, N., De Winter, T., Verbrugghe, K., Cleenwerck, I., Vandamme, P., Takrama, J. S., Vancanneyt, M. & De Vuyst, L. 2007 Dynamics and biodiversity of populations of lactic acid bacteria and acetic acid bacteria involved in spontaneous heap fermentation of cocoa beans in Ghana. *Applied and Environmental Microbiology*, **73**(6), 1809–1824. (doi:10.1128/AEM.02189-06)

12. Pereira, G. V. d. M., Magalhães, K. T., de Almeida, E. G., da Silva Coelho, I. & Schwan, R. F. 2013 Spontaneous cocoa bean fermentation carried out in a novel-design stainless steel tank: Influence on the dynamics of microbial populations and physical-chemical properties. *International Journal of Food Microbiology*, **161**(2), 121–133. (doi:10.1016/j.ijfoodmicro.2012.11.018)

13. Moreira, I. M. d. V., Miguel, M. G. d. C. P., Duarte, W. F., Dias, D. R. & Schwan, R. F. 2013 Microbial succession and the dynamics of metabolites and sugars during the fermentation of three different cocoa (*Theobroma cacao* L.) hybrids. *Food Research International*, **54**(1), 9–17. (doi:10.1016/j.foodres.2013.06.001)

14. De Vuyst, L. & Weckx, S. 2016 The cocoa bean fermentation process: from ecosystem analysis to starter culture development. *Journal of Applied Microbiology*, **121**(1), 5–17. (doi:10.1111/jam.13045)

15. Papalexandratou, Z., Vrancken, G., Bruyne, K. D., Vandamme, P. & Vuyst, L. D. 2011 Spontaneous organic cocoa bean box fermentations in Brazil are characterized by a restricted species diversity of lactic acid bacteria and acetic acid bacteria. *Food Microbiology*, **28**(7), 1326 – 1338. (doi:10.1016/j.fm.2011.06.003)

16. Vehtari, A., Gelman, A. & Gabry, J. 2017 Practical Bayesian model evaluation using leave-one-out cross-validation and WAIC. *Statistics and Computing*, **27**(5), 1413–1432. (doi:10.1007/s11222-016-9696-4)

17. Watanabe, S. 2010 Asymptotic Equivalence of Bayes Cross Validation and Widely Applicable Information Criterion in Singular Learning Theory. *Journal of Machine Learning Research*, **11**, 3571–3594.
